# Alleviating nanostructural phase impurities enhances the optoelectronic properties, device performance and stability of cesium-formamidinium metal–halide perovskites†

**DOI:** 10.1039/d4ee00901k

**Published:** 2024-05-09

**Authors:** Mostafa Othman, Quentin Jeangros, Daniel A. Jacobs, Moritz H. Futscher, Stefan Zeiske, Ardalan Armin, Anaël Jaffrès, Austin G. Kuba, Dmitry Chernyshov, Sandra Jenatsch, Simon Züfle, Beat Ruhstaller, Saba Tabean, Tom Wirtz, Santhana Eswara, Jiashang Zhao, Tom J. Savenije, Christophe Ballif, Christian M. Wolff, Aïcha Hessler-Wyser

**Affiliations:** a Ecole Polytechnique Fédérale de Lausanne (EPFL), Institute of Electrical and Micro Engineering (IEM) Photovoltaics and Thin-Film Electronics Laboratory (PV-Lab) Neuchâtel Switzerland mostafa.othman@epfl.ch christian.wolff@epfl.ch aicha.hessler@epfl.ch; b Centre d’Electronique et de Microtechnique (CSEM) Rue Jaquet-Droz 1 2000 Neuchâtel Switzerland; c Laboratory for Thin Films and Photovoltaics, Empa – Swiss Federal Laboratories for Materials Science and Technology Überlandstrasse 129 8600 Dübendorf Switzerland; d Sustainable Advanced Materials (Ser-SAM), Department of Physics, Swansea University Swansea SA2 8PP UK; e Advanced Instrumentation for Nano-Analytics (AINA), Luxembourg Institute of Science and Technology (LIST), Materials Research and Technology Department 41 Rue du Brill Belvaux L-4422 Luxembourg; f Swiss-Norwegian Beamlines at the European Synchrotron Radiation Facility 71 Avenue des Martyrs F-38000 Grenoble France; g Fluxim AG Katharina-Sulzer-Platz 2 Winterthur 8400 Switzerland; h Department of Chemical Engineering, Delft University of Technology Delft The Netherlands; i University of Luxembourg 2 Avenue de l’Université Esch-sur-Alzette L-4365 Luxembourg

## Abstract

The technique of alloying FA^+^ with Cs^+^ is often used to promote structural stabilization of the desirable α-FAPbI_3_ phase in halide perovskite devices. However, the precise mechanisms by which these alloying approaches improve the optoelectronic quality and enhance the stability have remained elusive. In this study, we advance that understanding by investigating the effect of cationic alloying in Cs_*x*_FA_1−*x*_PbI_3_ perovskite thin-films and solar-cell devices. Selected-area electron diffraction patterns combined with microwave conductivity measurements reveal that fine Cs^+^ tuning (Cs_0.15_FA_0.85_PbI_3_) leads to a minimization of stacking faults and an increase in the photoconductivity of the perovskite films. Ultra-sensitive external quantum efficiency, kelvin-probe force microscopy and photoluminescence quantum yield measurements demonstrate similar Urbach energy values, comparable surface potential fluctuations and marginal impact on radiative emission yields, respectively, irrespective of Cs content. Despite this, these nanoscopic defects appear to have a detrimental impact on inter-grains’/domains’ carrier transport, as evidenced by conductive-atomic force microscopy and corroborated by drastically reduced solar cell performance. Importantly, encapsulated Cs_0.15_FA_0.85_PbI_3_ devices show robust operational stability retaining 85% of the initial steady-state power conversion efficiency for 1400 hours under continuous 1 sun illumination at 35 °C, in open-circuit conditions. Our findings provide nuance to the famous defect tolerance of halide perovskites while providing solid evidence about the detrimental impact of these subtle structural imperfections on the long-term operational stability.

## Abstract 2

The impact of the composition of the A-site cations on the intra-domain defect density in cesium-formamidinium perovskite formulations is modulated *via* alloying.

## Graphical abstract

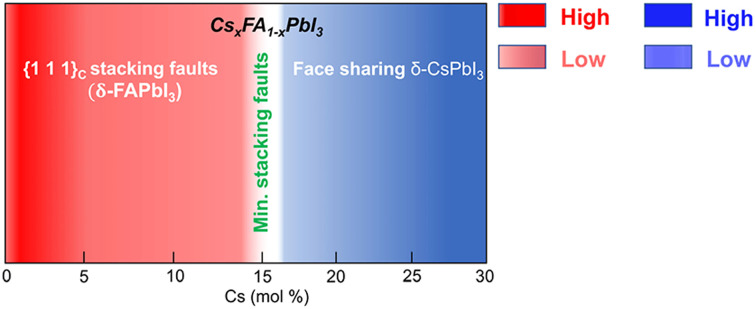

**Broader context**

Achieving record single-junction and tandem perovskite devices has been driven by compositional tuning of formamidinium-rich perovskite formulations. However, FAPbI_3_ is challenging to stabilize at room temperature as the desired α-FAPbI_3_ phase transitions to photoinactive wide-bandgap hexagonal polytypes. Macroscopically, alloying FA^+^ with Cs^+^ has proven to be a promising strategy for stabilizing cubic FAPbI_3_-like structures at room temperature. In this context, there is a lack of fundamental understanding of the stability mechanisms afforded by these alloyed approaches. Othman *et al.* studied the local structure in cesium–formamidinium perovskite absorbers using advanced nanoprobe techniques. They showed a variation in the crystallographic defect density with respect to the Cs/FA molar ratios and demonstrated that they are not sites of significant recombination losses. While they limit carriers’ transport and deteriorate the device's longevity.

## Main

The emergence of halide perovskite materials as a promising photovoltaic (PV) technology has revolutionized the solar energy research landscape.^1–3^ The rapid development of perovskite solar cells (PSCs) has been mainly driven by their potentially low-cost solution processibility and attractive optoelectronic properties.^4–6^ PV devices fabricated from perovskite absorbers have recently reached certified power conversion efficiency (PCE) values of more than 26% for single-junctions and 33% for tandem perovskite/silicon configurations.^7^ Efforts to realize metal–halide PSCs with record PCEs have been focusing on formamidinium-rich lead-iodide (FAPbI_3_; FA^+^ denotes formamidinium ion) perovskite formulations because of their near-ideal bandgap (∼1.5 eV) close to the maximum of the Shockley–Queisser limit (1.33 eV) and enhanced thermal stability compared to methylammonium (MA^+^)-based perovskites.^8–12^ However, retaining such performance under operational conditions remains elusive as FAPbI_3_ suffers from structural instability where the desired photo-active α-FAPbI_3_ cubic phase reverts to a non-perovskite wide-bandgap photo-inactive δ-FAPbI_3_ phase at room temperature.^13,14^

Various strategies have been adopted to suppress the formation of the δ-FAPbI_3_ phase, notably cesium (Cs^+^) alloying with FA^+^ on the A-site of the perovskite crystal structure.^15–17^ Cesium–formamidinium (CsFA) cation mixing has demonstrated improved cubic perovskite structure stability by tuning the so-called Goldschmidt tolerance factor (*t*) towards a value of *t* ∼ 1, which empirically forms a stable cubic perovskite phase.^18^ This stabilization has been rationalized by first-principle calculations claiming that A-site mixing results in entropic gains which lowers the free energy required to form the cubic phase, whereas on the contrary, the δ-phase formation energy becomes too large to overcome the configurational entropy increase.^19^ Interrogating the stability behaviour established by CsFA alloying has been reliant on either theoretical studies or bulk-averaged macroscopic measurements.^20^ By contrast, little effort has been devoted to understand the stability mechanisms driven by the CsFA alloyed approach from a nanoscopic point of view. In this context, there is a lack of fundamental studies to characterize the local structure of CsFA perovskite absorbers compared to their triple-cations (CsMAFA) counterparts.^21–24^ The reasons are manifold: generally, probing the perovskite nanostructure requires the use of transmission electron microscopy (TEM) techniques operating with high energy electrons (200 keV) where the beam-sensitive nature of hybrid perovskites makes it extremely challenging to obtain reliable local structural information from the pristine material.^25^ Additionally, CsFA is challenging to fabricate in high crystal quality and compact morphology without optimized precursor solution ageing combined with the use of hydrohalic acids as additives; an experimental methodology that is incompatible to reproduce and imposes constraints on the process feasibility.^26–28^ Lastly, there have been a few reports mentioning complexities of CsFA elemental mixing at the solution stage as well as uncertainty of compositional control in the final thin film due to the presence of inevitable phase separation when Cs content is >30 mol% for the latter and low solubility of the inorganic Cs salts in solvents for the former, which render this perovskite formulation as disadvantageous and ultimately hampers its deployment in solar cell devices.^29–31^ To mitigate these issues, a small amount of MA^+^ is typically added to CsFA perovskite precursors to aid in the crystallization of a high-quality film. On the downside, volatile MA^+^ can decompose readily and initiate degradation during device operation.^32–35^

Stimulated by the challenging nature of CsFA perovskite absorbers and motivated in probing the intrinsic stabilization mechanisms induced by cation alloying, in this work, we unravel the underpinning local structural evolution in Cs_*x*_FA_1−*x*_PbI_3_ perovskites and assess its influence on the optoelectronic properties, device performance and operational stability. We find a compositional dependent correlation between the nanoscopic defect nature and Cs/FA molar ratios. We further evaluate the effect of these structural defects on the optoelectronic quality of the perovskite films and devices. We show that these structural defects are not sites of significant recombination losses, while on the other hand, they appear to impede carriers transport in devices. Finally, we demonstrate that trace amounts of these structural defects, undetected macroscopically, lead to compromised stability in the long-term testing under operational conditions. Our results construct a global picture of the nanoscopic landscape, which endows the defect tolerance of perovskite materials to structural disorders, while highlighting the detrimental impact of these nanoscale phase impurities on limiting the device longevity.

## Results and discussion

**Fig. 1:**
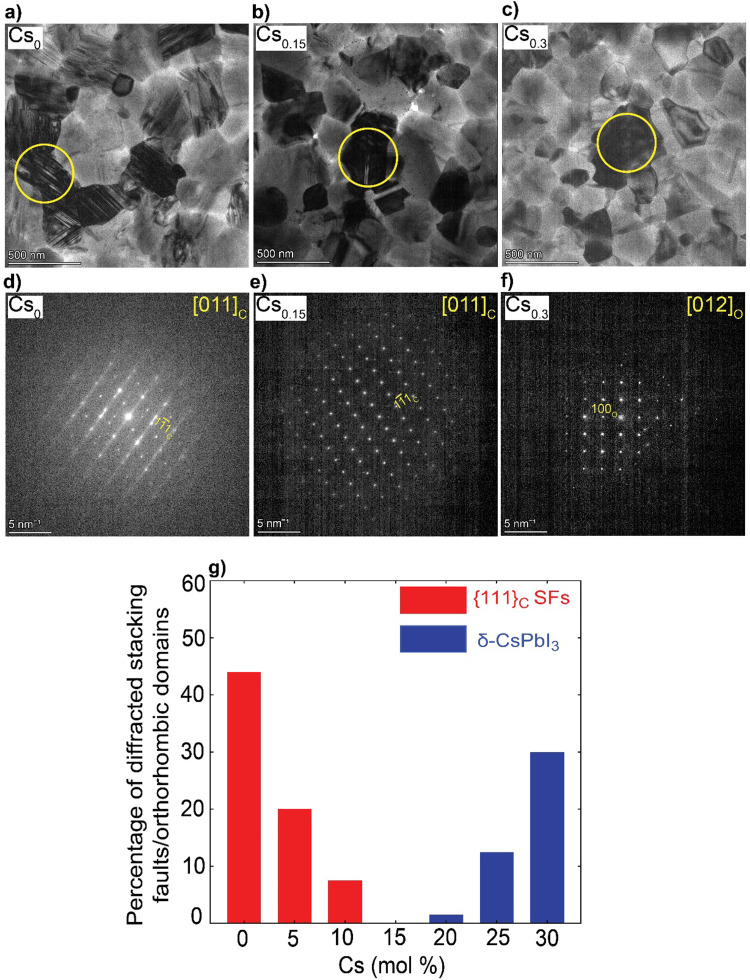
Nanostructural analysis of the Cs_*x*_FA_1−*x*_PbI_3_ perovskite films: (a)–(c) BF micrographs, (d)–(f) The associated SAED patterns (The Cs content and zone axis are written on the top left and right side of the DPs, respectively), The yellow circles indicate the position of the selected-area for SAED pattern acquisition; and (g) Histograms illustrating the impact of Cs content on the defect density. The estimated percentages are based on the number of diffracted domains (from all the obtained micrographs) featuring {111}_C_ SFs or δ-CsPbI_3_ phases with respect to all the other different phases present in the samples.

We solution-processed mixed-cation Cs_*x*_FA_1−*x*_PbI_3_ (where *x* = 0, 0.05, 0.1, 0.15, 0.2, 0.25, 0.3; note that the concentrations are nominal, derived from the compositions in the precursor inks) perovskite absorbers on ultra-thin carbon (C)-coated copper (Cu) TEM grids. For the deposition of electron-transparent films (<200 nm in thickness), we used diluted ink concentrations while keeping the same solvents and processing conditions used for solar cell fabrication to enable sample representation. First, we estimated the *t* values using the formula 
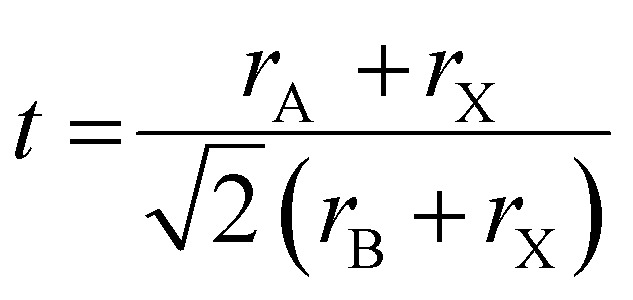
 (where *r*_A_, *r*_B_ and *r*_X_ are ionic radii of the A, B and X-site ions in the ABX_3_ perovskite structure^36^) for the Cs_*x*_FA_1−*x*_PbI_3_ perovskite thin-films, to be used as a rough guide for predicting the perovskite structural phase stability (*t* ∼ 1) Table S1 (ESI†). Varying the Cs/FA molar ratios yields different *t* values, which is expected to have a significant impact on the local structure formation and defect-density. Furthermore, to mitigate beam-damage induced artifacts, the total TEM dose was minimized. We used an electron dose rate of ∼1 e Å^−2^ s^−1^ for our TEM imaging and diffraction conditions. Based on our results, we found total doses above ∼7 e Å^−2^ led to a quick degradation of the material (ESI,† Fig. S1 and Videos S1, S2), in agreements with the reported beam-damage doses for FAPbI_3_^37^ and as a result, our imaging and diffraction conditions were recorded below those values. To study the evolution of crystallographic defects in Cs_*x*_FA_1−*x*_PbI_3_ perovskite films, we collected low-dose bright field (BF) TEM micrographs and selected-area electron diffraction (SAED) patterns extracted locally from individual domains (Fig. 1). The TEM micrograph of FAPbI_3_ (Cs_0_) reveals a prevalence of observable stacking faults (SFs) in striped contrast with widths between 5 to 7 nm in many domains all over the film surface (Fig. 1(a)). An SAED pattern extracted from the domain highlighted with the yellow circle in Fig. 1(a) displays the {111}_C_ twin domains oriented near the [011]_C_ zone axis of the metrically cubic FAPbI_3_ perovskite *Pm*3̄*m* crystal structure with a lattice parameter of 6.3 Å (Fig. 1(d)). Note that, throughout the manuscript, the subscripts “c”, “h” and “o” stands for an indexation in the cubic, hexagonal and orthorhombic phases, respectively. Importantly, we observe quantitively a gradual reduction in the {111}_C_ SFs and twin densities with increasing the Cs molar content, (at Cs_0.05_ & Cs_0.1_ as shown in ESI,† Fig. S2a, d, b and e) as evident in the BF micrographs and their associated diffraction patterns (DPs). The formation of the {111}_C_ SFs is likely attributed to heterogeneous A-site cation (Cs^+^ to FA^+^) distributions, which would allow microscopically unstable regions to form inside the cubic perovskite domains such as the hexagonal photo-inactive δ-FAPbI_3_ phase. To verify this postulation, helium ion microscopy with secondary ion mass spectrometry (HIM–SIMS) imaging was performed to probe the elemental distribution in CsFA perovskite films. As can be seen in ESI,† Fig. S6a–o, the Cs, Pb, C, N and I elemental mapping shows relatively uniform distribution in the Cs_0.15_FA_0.85_PbI_3_ perovskite absorber (with the exception of Pb–I rich clusters, likely PbI_2_) compared to the FAPbI_3_ and Cs_0.3_FA_0.7_PbI_3_ perovskite films which agrees with our hypothesis. Note that, the slightly high signal on the left half of the Cs map in ESI,† Fig. S6h is an artifact, which could be attributed to minor variations in the secondary ion extraction efficiencies during the experiment.

Interestingly, no SFs were detected in Cs_0.15_FA_0.85_PbI_3_ perovskite absorbers in all the metrically cubic structures near the [011]_C_ zone axis (from an analysis of 25 diffraction patterns (DPs) that were obtained across multiple batches and experiments) (Fig. 1(b), (e) and ESI,† Fig. S3a–d). A similar observation was reported by Li *et al.* for MA_0.8_FA_0.2_ when tuning the composition of MA_*x*_FA_1−*x*_PbI_3_ perovskite films.^37^ A further increase in Cs content (*x* = 0.2–0.3) results in the appearance of unwanted Cs-rich non-perovskite secondary phases known as δ-CsPbI_3_ as presented in Fig. 1(c), (f) and ESI,† Fig. S2c, f, g and h, Note S1. On the basis of these observations, we conclude that Cs alloying changes the intrinsic FAPbI_3_ perovskite defect density in which low Cs concentrations (Cs < 15 mol%) reduce the {111}_C_ SFs with a minimum reached at Cs_0.15_. On the other hand, the presence of CsFA substitutional alloying limit as well as elemental mixing complexities in the precursor stage with high-Cs content (Cs > 15 mol%) tend to trigger the formation of segregated Cs-rich δ-CsPbI_3_ clusters in the perovskite films.^29–31^ Therefore, there is a compositional dependence of structural defects prevalence and Cs/FA molar ratios (Fig. 1(g)). We note that we did not observe any single domain that could be indexed with the δ-FAPbI_3_ crystal structure (2H or even the polytypes 4H and 6H^14^) in all of the analysed Cs_*x*_FA_1−*x*_PbI_3_ films, which suggests that single-halide systems have a lower density of bulk hexagonal polytypes compared to mixed-halides.^21^ For all the Cs-alloyed films, kinematically forbidden reflections in the cubic phase were detected. These can only be indexed to a cubic superstructure with the *Im*3̄ space group near the [011]_C_ zone axis (ESI,† Fig. S4).^38^ Recently, Doherty *et al.* studied these forbidden reflections in similar FA-rich perovskite compositions such as CsMAFA, attributing them to octahedral tilting whereby the BX_6_ corner-sharing octahedra slightly tilt away from a perfect cubic symmetry in a long-range ordered fashion.^22^ The unit cell parameter of the Im3̄ space group is effectively doubled (∼12.6 Å) with the structural ordering of the lattice tilting, leading to so-called superlattice reflections. However, since we did not observe any of these superstructure reflections in the neat FAPbI_3_ films from a study of hundreds of DPs, we cannot exclude the possibility of CsFA cation ordering on the A-site as a driving force for these superlattice reflections in these alloyed systems. Moreover, upon *in situ* annealing of a pure FAPbI_3_ film inside the TEM at 170 °C, we detected the emergence of hexagonal polytype phases with a domain that could be indexed to the 6H-FAPbI_3_ phase along the [372̄]_H_ zone axis as highlighted in ESI,† Fig. S5. This observation indicates the thermal decomposition of the metrically cubic FAPbI_3_ film is possibly due to FA^+^ loss which results in a change of the corner-sharing stacking sequence (metrically cubic perovskite) to a mix of corner-face sharing octahedra (hexagonal polytypes) when annealed at elevated temperatures. Prolonged heating under vacuum should eventually lead to lattice shrinkage, morphology deformation and degradation of the perovskite film.^39^

**Fig. 2:**
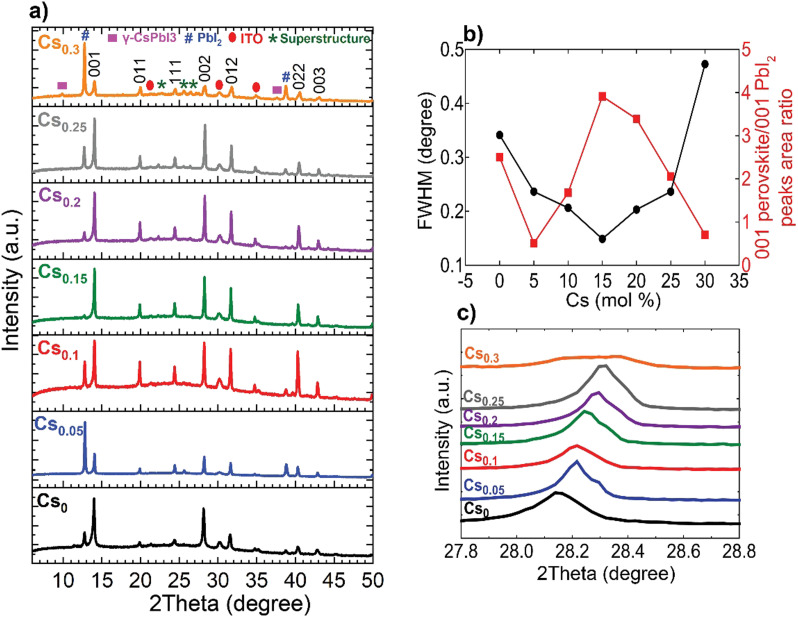
Macroscopic structural analysis of the Cs_*x*_FA_1−*x*_PbI_3_ perovskite films: (a) XRD patterns, (b) Analysis of the FWHM of the 001 perovskite peak and relative 001 perovskite/001 PbI_2_ as a function of the Cs content and (c) zoomed-in view of the 002 perovskite peak highlighting shift to higher angles with increasing Cs content indicative of a lattice contraction.

The perovskite films were examined macroscopically using X-ray diffraction (XRD) measurements, shown in Fig. 2(a). The 001, 011, 111, 002, 012, 022 and 003 of the metrically α-cubic FAPbI_3_ perovskite structure in addition to peaks corresponding to PbI_2_ and the ITO substrate are present. Although, the inks containing 10 mol% excess PbI_2_ for all compositions, which is reported to improve efficiency,^40^ we noticed a change in the relative 001 PbI_2_ to 001 perovskite peak intensities located at 12.67° and ∼14°, respectively. We rationalize this observation by the likelihood of the existence of definite packaging density of Cs-ions relative to FA^+^ on the A-site of the perovskite lattice to form the metrically cubic phase where the threshold is found to be at Cs_0.15_. Upon exceeding this limit (Cs > 15 mol%) and with the oversupply of Cs-ions, the perovskite lattice starts to be likely strained and gradually decomposes into PbI_2_.^41^ The Cs_0.15_FA_0.85_PbI_3_ perovskite film exhibits the smallest full-width at half maximum (FWHM) for the main 001 perovskite peak in addition to the highest relative intensity of 001 perovskite to 001 PbI_2_ peaks, inferring enhanced crystalline ordering of the perovskite lattice (Fig. 2(b)). Furthermore, the incorporation of the smaller-sized Cs cation into the FAPbI_3_ crystal lattice leads to a shift to higher angles for the 002 perovskite peak, implying smaller *d*-spacings and hence, lattice contraction (Fig. 2(c)). For the Cs_0.3_FA_0.7_ films, we observe a peak broadening and a disruption of the aforementioned trend where the peaks do not stagnate with respect to width and position, but are broader and weaklier defined, indicating unsuccessful Cs-incorporation and actual disruption of the crystal lattice symmetry at these higher concentrations. Additionally, HIM–SIMS measurements (ESI,† Fig. S6k–o) demonstrate the distinct formation of segregated CsPbI_3_-rich clusters. These observations are consistent with the previous reports about the presence of a Cs-substitution limit in CsFA alloying.^30^

Morphologically, the perovskite surface of the FAPbI_3_ film consists of rough (verified by atomic-force microscopy (AFM) in ESI,† Fig. S8) terraced-edges, which are likely SFs based on our TEM data, as corroborated by scanning electron microscopy (SEM) images in ESI,† Fig. S7a. In stark contrast, the CsFA alloyed films display well-defined domains and a decrease in domain sizes relative to FAPbI_3_ films with increasing Cs content (ESI,† Fig. S7b–g). Here, we refer our discussion to an overlooked aspect in perovskite literature: domain crystal quality rather than domain size. It is widely accepted that growing large grains with fewer grain boundaries is beneficial for charge transport and thereby, gaining efficiency improvements.^42–44^ However, it was recently reported that SEM observations are usually based on morphological domains comprised of multiple grain entities rather than being singular grains themselves and that the domains’ local orientations are much more critical than their sizes.^45^ In concert with these observations, we found that Cs_0.15_FA_0.85_PbI_3_ films possess relatively smaller-domain sizes but are SF-minimal indicating higher structural quality. Meanwhile, low Cs-content (Cs_0.05_FA_0.95_PbI_3_ and Cs_0.1_FA_0.9_PbI_3_, respectively) alloyed films consist of larger domains with considerably higher densities of SFs. Therefore, this calls for diverting efforts into the promotion of the domain quality through the elimination of structural disorders rather than seeking to only enhance the domain size. Needless to say, an ideal growth condition entails creating large domains with high crystal quality perovskite films.

**Fig. 3:**
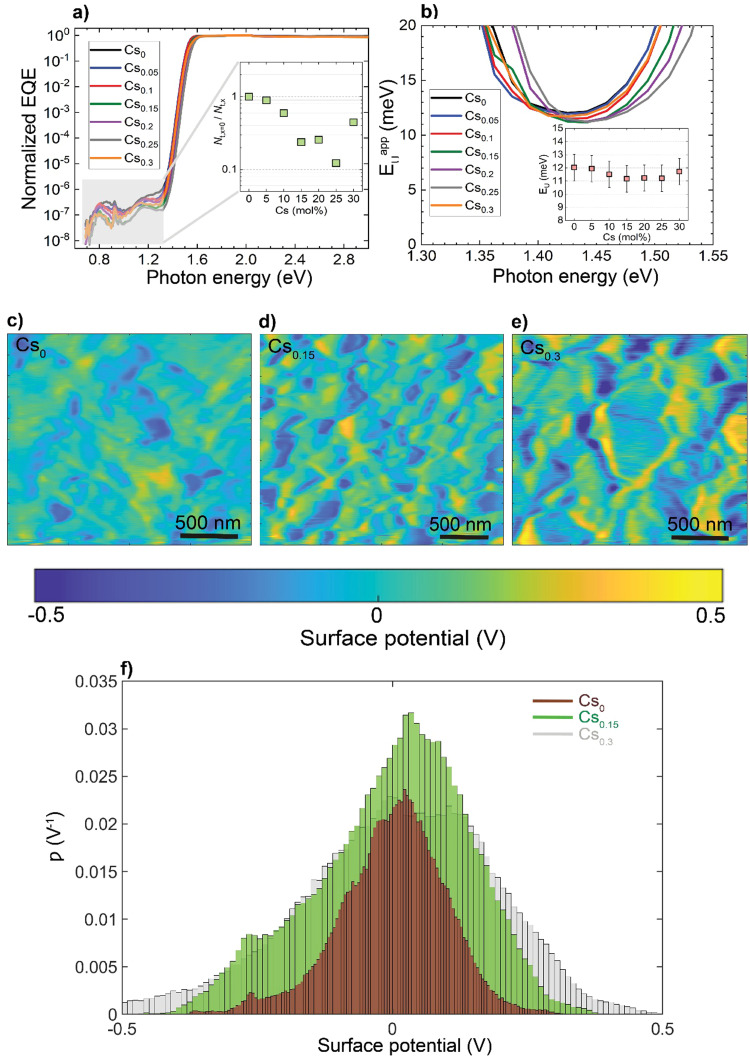
The impact of Cs alloying on electronic trap states: (a) normalized EQE of Cs_*x*_FA_1−*x*_PbI_3_ perovskite devices plotted as a function of photon energy. The inset shows the change of deep trap signal with Cs content as obtained from the integral of EQE in the trap-limited area *versus* Cs fraction in Cs_*x*_FA_1−*x*_PbI_3_ perovskite devices, (b) apparent Urbach energy spectra of the devices plotted as a function of photon energy. The inset shows the Urbach energy spectrum against the Cs concentration with error bars corresponding to the ±1 meV uncertainty in Urbach energy determination, (c)–(e) Surface potential maps for the CsFA perovskite films and (f) corresponding distribution (*ρ*) plots of the relative surface potential shifts for the perovskite films.

To investigate how these structural and morphological features influence the films’ optoelectronic properties, we first sought to address whether those nanoscale defects contribute electronically to shallow or deep trap states. To test for the presence of shallow traps, we performed ultra-sensitive external quantum efficiency (EQE), kelvin-probe force microscopy (KPFM) measurements (Fig. 3). Ultra-sensitive EQE measurements were conducted on encapsulated Cs_*x*_FA_1−*x*_PbI_3_ perovskite devices using a home-built sensitive EQE apparatus.^46^Fig. 3(a) shows the normalized EQE spectra of Cs_*x*_FA_1−*x*_PbI_3_ devices plotted as a function of photon energy, and compared for different concentrations of Cs. We note that the sharp peak located at approximately 0.92 eV is electrical device- and/or EQE apparatus-related pick-up noise since the genuine spectral features in the EQE are never as sharp as this 0.92 eV peak.^47^ On the other hand, the line-shape of all perovskite EQE spectra is dominated by the 0.85 eV and 1.2 eV interference peaks, which includes an additional, Cs-concentration dependent, mid-gap trap signal (see inset in Fig. 3(a)), this is expected and in line with the literature.^48^ As shown from the integrated sub-gap EQE (inset in Fig. 3(a)), where the integrated area can be treated as rough measure for relative changes in trap signal/density (under the condition that the device thickness is the same, hence, interference effects are the same), with a reduction upon Cs-alloying, likely linked to improved performance through reduced recombination losses.^49,50^ The apparent Urbach energy spectra,^51^
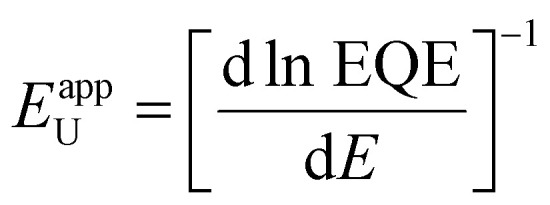
, were calculated based on the normalized EQE spectra, and are shown in Fig. 3(b). Here, the Urbach energy is found to be approximately 11.5 ± 1 meV for all perovskite devices (inset in Fig. 3(b)) indicative of the absence of Cs-induced changes in shallow trap (or tail) states.^47^ We further tracked the impact of Cs alloying on the surface potential distributions using KPFM. It is worth noting that surface defects can modify the potential distributions of perovskite surfaces.^52^ Since these measurements were done under ambient conditions and subsequently, consecutive measurements on the same scan area may not always yield the same absolute values, we focused our interpretation only on surface potential variations (degree of homogeneity) and not absolute potential values. As shown in Fig. 3(c)–(e) and ESI,† Fig. S9a–d, the surface potential fluctuations of all perovskite films were comparable regardless of Cs content. The standard deviations of surface potential distributions are presented in Fig. 3(f) and the electronic-roughness values are compared in ESI,† Fig. S9e. To verify our surface potential findings and exclude the possibility of any topographical features induced artifacts (such as valleys present at grain boundaries that might not be detected by the tip), we plotted correlation maps between the surface topography and potential values as shown in ESI,† Fig. S10a–g. We then were able to firmly conclude that they are not correlated with very low correlation factors (*R*) < 20%, confirming the soundness of our experimental results (ESI,† Fig. S10h). Likewise, there is no signature of differences in surface potential mimicking the shape of the observed striped contrasts due to stacking faults. This means that the observed potential variations do not reflect directly in a change in surface potential.

Furthermore, measurements of photoluminescence quantum yield (PLQY) *versus* Cs concentration show only a minor effect of Cs content on the film-averaged non-radiative recombination rate at 1-sun illumination intensity (ESI,† Fig. S11). Taken together, these measurements suggest that these structural defects are not inducing either surface or deep traps and hence, are not sites of significant recombination losses.

Applying temperature-dependent ultra-sensitive EQE measurements on unencapsulated Cs_*x*_FA_1−*x*_PbI_3_ PSCs, we were able to probe the dynamic (*E*_dyn_) and static (*E*_stat_) energetic disorder.^53^ The latter has been shown to be intrinsically related to zero point motion of phonons rather than structural imperfections,^47,54^ while the former is a metric for temperature-dependent phonon occupation (proportional to phonon energy).^55^ We note that the temperature regime (between 25 °C to −120 °C) was chosen such that phase-transition of the main perovskite structure (*i.e.*, FAPbI_3_) at the higher and lower temperature limits were avoided.^52^ ESI,† Fig. S12a–g displays the normalized EQE of the Cs_*x*_FA_1−*x*_PbI_3_ perovskite devices (*x* = 0, 0.5, 0.1, 0.15, 0.2, 0.25, and 0.3, respectively) plotted as a function of photon energy and compared against different temperatures. The insets show the corresponding calculated apparent Urbach energy spectra, from which the Urbach energies were determined. The Urbach energy for the Cs_*x*_FA_1−*x*_PbI_3_ perovskite devices at room temperature were found to be 12.1 ± 1 meV (*x* = 0), 11.5 ± 1 meV (*x* = 0.05), 11.8 ± 1 meV (*x* = 0.1), 11.0 ± 1 meV (*x* = 0.15), 11.5 ± 1 meV (*x* = 0.2), 11.9 ± 1 meV (*x* = 0.25), and 11.5 ± 1 meV (*x* = 0.3). These values are in good agreement with Urbach energy values obtained on encapsulated devices in inset Fig. 3(b). ESI,† Fig. S13a–g shows Urbach energy values plotted against the temperatures and compared for all the Cs_*x*_FA_1−*x*_PbI_3_ PSCs. While symbols are experimental data, black solid lines are fits to the Einstein solid model: 
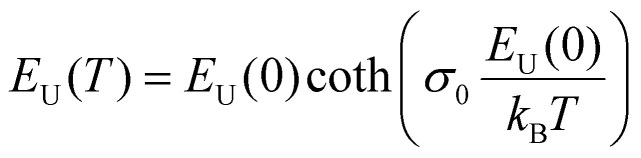
, where 
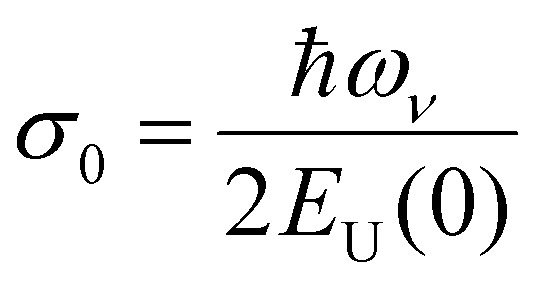
 is a temperature-independent constant, *ω*_*v*_ is a characteristic phonon frequency, ℏ is the reduced Planck constant, and *k*_*B*_ is the Boltzmann constant.^47^ Based on the Einstein solid model, temperature-induced *E*_dyn_ and *E*_0_ disorder contribute to Urbach energy *E*_U_(*T*), *via E*_U_(*T*) = [*E*_0_ + *E*_dyn_]/*σ*_0_, where *E*_0_ is expected to dominate at the low-temperature limit (*i.e.*, for *T* → 0, *E*_U_(*T*) →*E*_0_). *E*_0_ (defined here as phonon zero-point energy) is found to be 3.26 ± 0.24 meV (*x* = 0), 3.82 ± 0.09 meV (*x* = 0.05), 3.75 ± 0.15 meV (*x* = 0.1), 4.55 ± 0.18 meV (*x* = 0.15), 5.39 ± 0.09 meV (*x* = 0.2), 6.68 ± 0.13 meV (*x* = 0.25), and 4.00 ± 0.15 meV (*x* = 0.3). The Urbach energy at room temperature, *E*_U_(RT), the Einstein solid mode fit parameter *E*_0_ and *σ*_0_, and the calculated approximate Einstein temperature, 
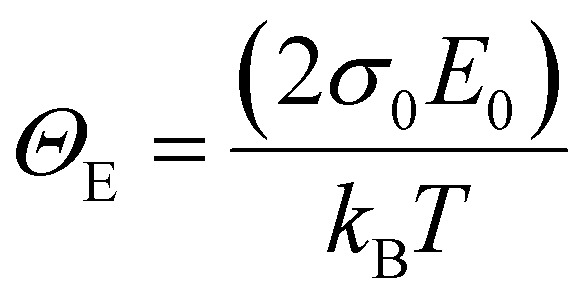
, and phonon wavenumber are provided in Table S2. The corresponding calculated wavenumbers match very well to Pb–I octahedral and cage-cation phonon modes, respectively, as obtained from Raman spectroscopy, reported in the literature.^53–55^ While the Einstein solid model can be extended by introducing an additional term *E*_struc_ to account for, *inter alia*, defect state-induced structural disorder, however, only for *E*_struc_ = 0 reliable fits of *E*_U_(*T*) for the Cs_*x*_FA_1−*x*_PbI_3_ perovskite devices were achieved. The absence of an additional structural disorder in these materials suggests that static disorder is solely dominated by quantum mechanical phonon lattice vibrations (phonon zero-point energy).^49^ This indicates that the introduction of Cs atoms in the perovskite lattice does not impose structural deformations as our data can be explained by a simple Einstein solid model with no need for an additional structural disorder as expected for example with amorphous silicon (a-Si),^47^ which is in accordance with our observation of almost no changes in Urbach energy with Cs doping.

**Fig. 4:**
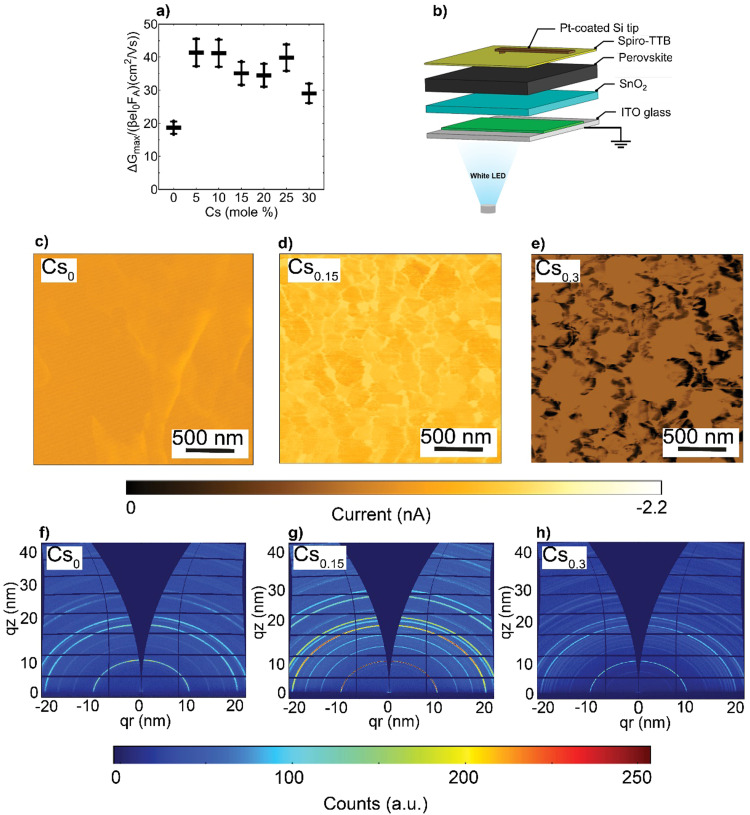
Optoelectronic properties and crystal orientation: (a) charge carrier mobility values calculated from TRMC measurements with error bars corresponding to 10% uncertainty, (b) Experimental setup of the C-AFM measurements where a conductive tip is functioning in contact mode, (c)–(e) Photo-current maps performed at 1 sun under *J*_SC_ conditions for the CsFA perovskite systems and (f)–(h) 2D GIWAXS patterns of the CsFA perovskite films.

Next, to investigate the effects of the nanoscale defects on the photo-conductivity of the CsFA perovskite films, we performed time-resolved microwave conductivity (TRMC) measurements and observed a comparable increase of carriers’ mobilities in the range of 30–40 cm^2^ (V s)^−1^ upon Cs introduction, except for the Cs_0_ as shown in Fig. 4(a). Notably, these values present intra-grain/domain “within the internal structure of the grain itself” mobilities, yet barriers at the edges of domains may result in effective reduced mobilities throughout the film.^56^ We further note that the TRMC traces are recorded at an incident light intensity of 5.5 × 10^9^ photons per cm^2^ per pulse, which corresponds to an initial charge carrier density of approximately 10^14^ cm^3^ per pulse. Complementarily, we conducted impedance spectroscopy and potentiostatic polarization measurements (see ESI,† Note S2 for details),^57,58^ and observed that the electronic conductivity (*σ*_electronic_) is maximized in the Cs_0.15_FA_0.85_PbI_3_ perovskite film, while the ionic conductivities (*σ*_ionic_) decrease with increasing Cs content (ESI,† Fig. S14a and b). We suspect that this change in ionic conductivity is related to a change in lattice constant upon Cs incorporation (Fig. 2(c)). It has been shown that compression of the lattice reduces ion migration by increasing the associated activation energy.^59^ In addition, we find that the ion densities (*n*_ion_) remain almost constant with increasing Cs content (ESI,† Fig. S14c). As a result, the change in ionic conductivity is probably related to a change in the ion mobility.

**Fig. 5:**
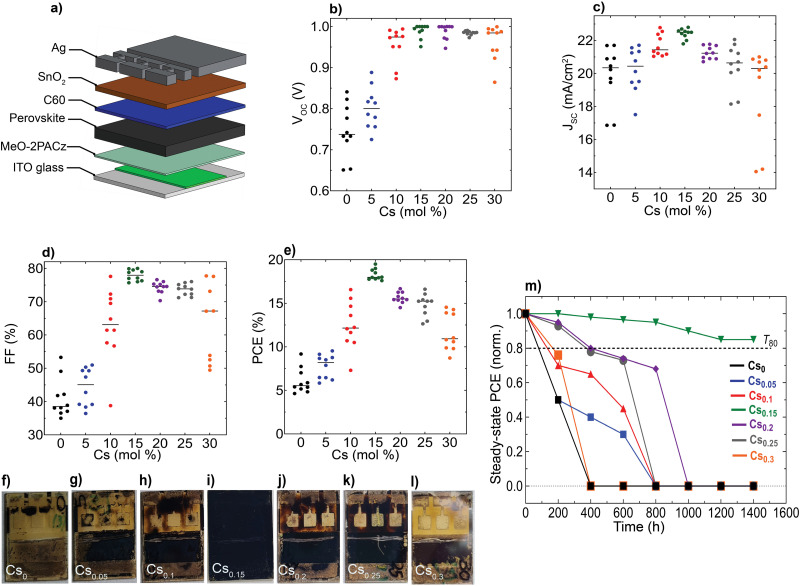
Device performance and operational stability of the perovskite devices: (a) schematic illustration of the pin device stack, Statistical distribution for the device performance metrics of the CsFA PSCs: (b) *V*_OC_, (c) *J*_SC_, (d) FF, (e) PCE. All *JV* data were acquired at standard conditions under a simulated air mass (AM) 1.5G and 100 mW cm^−2^ of sunlight, (f)–(l) Photographs of the aged CsFA encapsulated perovskite devices taken from the glass side after 1400 hours of continuous simulated sunlight illumination and (m) Evolution of normalized (norm.) steady-state PCE (averaged from 6 sub cells) for encapsulated PSCs, aged under continuous simulated sunlight illumination at 35 °C in N_2_ atmosphere, *T*_80_ defines the time required for a solar cell device to drop to 80% of its initial efficiency.

To evaluate the local photo-current at a nanoscale, conductive-atomic force microscopy (C-AFM) was used with a white LED providing ∼1 sun excitation, as shown in Fig. 4(b). The energy-levels diagram for the different layers comprising the pseudo-device stack and the work function (WF) of the platinum (Pt)-coated silicon (Si) tip are depicted in ESI,† Fig. S15, based on prototypical literature values.^60–62^ Note, to enable local information, we refrained from depositing metal electrodes, which would collect charge-carriers from a larger area due to their high lateral conductivity, *i.e.*, reducing spatial resolution. As can be seen in Fig. 4(c)–(e), the spatial photo-current conductivity and homogeneity are peaked in Cs_0.15_FA_0.85_PbI_3_ perovskite system compared to the Cs_0_ and Cs_0.3_ pseudo-devices, indicative of effective carriers’ transport and collection properties. This trend agrees with the short-circuit current (*J*_SC_) obtained from full devices (presented later in Fig. 5(c)) with an *R* = 0.63 in ESI,† Fig. S16. These results provide an indication that nanoscale defects rather than charge carriers’ mobilities retard the charge carrier transport within the CsFA absorber layers. Therefore, we can conclude that the increased conductivity in Cs_0.15_FA_0.85_PbI_3_ is more likely attributed to the high crystal perovskite film quality. Moreover, although the SFs/Cs-rich clusters do not reduce the intra-grain carrier mobility, they seem to affect the inter-grain (across a set of individual grains) transport, as evidenced by C-AFM measurements.

Since the photo-electric properties such as *J*_SC_ of metal–halide perovskites vary greatly among different facets,^63–65^ we used 2D synchrotron grazing-incidence wide-angle X-ray scattering (GIWAXS) to probe the in- and out-of plane films’ texture. As shown in Fig. 4(f)–(h) and ESI,† Fig. S17a–d, theCs_*x*_FA_1−*x*_PbI_3_ (*x* = 0.05, 0.1, 0.15, 0.2 and 0.25, respectively) perovskite films display similar diffraction rings in the 001 and 002 main perovskite peaks at *q* about 10 and 20 nm^−1^, respectively. Notably, upon Cs alloying (pronounced even at Cs_0.05_), the random azimuthal orientation observed in the FAPbI_3_ film shifts to a mild preferential 001 face-up orientation^66^ relative to the substrate coinciding with a corner-up orientation of the 011 perovskite plane (ESI,† Fig. S18a–d). This plausibly further supports that the SFs/Cs-rich defective phases impose transport losses rather than the crystal orientation.

We aimed to investigate the impact of the differences observed in nanostructure and optoelectronic properties on the device performance and long-term operational stability for different Cs content inCs_*x*_FA_1−*x*_PbI_3_ PSCs, while keeping the hole/electron transport layers and device configuration the same. We fabricated devices using an inverted (p–i–n) architecture based on indium-tin oxide (ITO) glass/(2-(3,6-dimethoxy-9*H*-carbazol-9-yl)ethyl)phosphonic acid (MeO-2PACz)/perovskite/buckminsterfullerene (C_60_)/tin(iv)oxide (SnO_2_)/silver (Ag), as sketched in Fig. 5(a). Remarkably, the Cs_0.15_FA_0.85_PbI_3_ perovskite film reached a maximum PCE of 19.3% with a *J*_SC_ of 22.8 mA cm^−2^, an open-circuit voltage (*V*_OC_) of 1.02 V and a fill-factor (FF) of 82.8% (Fig. 5(b)–(e)). We further attempted to link the device data to the films’ optoelectronics. First, we performed a performance loss-breakdown analysis by extracting the quasi-Fermi level splitting (QFLS) for different charge-transport layers (CTLs)/perovskite combinations in comparison to the mean qV_OC_ of the devices. The losses are comparable for all stacks, however, we noticed major differences between the obtained *qV*_OC_ and QFLS for the poor-performing (notably Cs_0_ and Cs_0.05_) devices, as shown in ESI,† Fig. S19. We sought to understand this effect with a link to carriers’ mobility and therefore, conducted a set of drift-diffusion simulations rendering qualitatively a similar effect with respect to the *V*_OC_*versus* QFLS (albeit significantly less severe) and likewise a drop in the other PV metrics below a mobility of 1 cm^2^ V^−1^ s^−1^, as shown in ESI,† Fig. S20. The qualitative similarity between the QFLS/*V*_OC_ trend is in perfect agreement with a recent study by Warby *et al.*,^67^ yet there is a significant quantitative disagreement. We tried to understand this disagreement in greater detail and re-examined the current–voltage (*JV*) statistics and found that the missing link is the systematically lower apparent shunt resistance in the cells with Cs-content <15% contributing to a lower *V*_OC_, FF and *J*_SC_ in addition to the other detrimental effects due to the unfavourable nanostructure properties (ESI,† Fig. S21). Thus, we point out that the poorer intrinsic nano-structural quality of the perovskites with various Cs content (except Cs_0.15_FA_0.85_PbI_3_) together with a lower apparent shunt resistance account for the observed effects on device performances. Notably, we extracted the apparent shunt resistance as a function of illumination intensity and observed a strong intensity-dependence, in particular for the lowest-performing Cs_0_ samples (ESI,† Fig. S22). A classical electrical shunt (*i.e.*, direct connection of the ITO to Ag) would not have such an intensity-dependence. In contrast, impaired charge collection, *e.g.*, through a reduced mobility of at least one of the charge carriers, results in such a behaviour, more commonly observed in, *e.g.*, organic or similarly excitonic solar cells. These observations are in full accord with results by Elmestekawy *et al.*,^68,69^ who show a comparable reduction of intrinsic quantum confinement in FAPbI_3_ upon Cs substitution and discuss the impact of reduced confinement on device performance. Our investigations complement these observations *via* a nanoscopic investigation where the origin of said confinement is likely the nanoscopically separated domains emerging from the shown SFs and in excellent agreement with the predictions by Wright *et al.*^70^ Voltage-dependent EQE measurements suggest that primarily the hole transport is limited in the case of low Cs substitution (ESI,† Fig. S23).

Furthermore, we aged unencapsulated perovskite absorbers at constant simulated solar spectrum illumination in ambient atmosphere without an ultraviolet (UV) filter at ∼65 °C (ESI,† Fig. S24). In parallel, encapsulated devices (with cover slip and UV-cured glue) were aged under continuous full-spectrum of 1 sun illumination at 35 °C and open-circuit conditions in a nitrogen (N_2_) environment (Fig. 5(m) and ESI,† Fig. S25). We note that ageing PSCs at open-circuit conditions is reported to accelerate degradation compared to maximum power point tracking (MPP).^71^ Notably, the optimum Cs_0.15_FA_0.85_PbI_3_ perovskite device sustained 85% of its initial PCE for 1400 hours, whereas all the other devices completely degraded within comparatively different and shorter timescales, consistent with their yellowish-visual appearances (Fig. 5(f)–(l)), particularly in the device-active area regions which is indicative of PbI_2_ phase formation generated as a by-product of the absorber layers decomposition.^72^

**Fig. 6:**
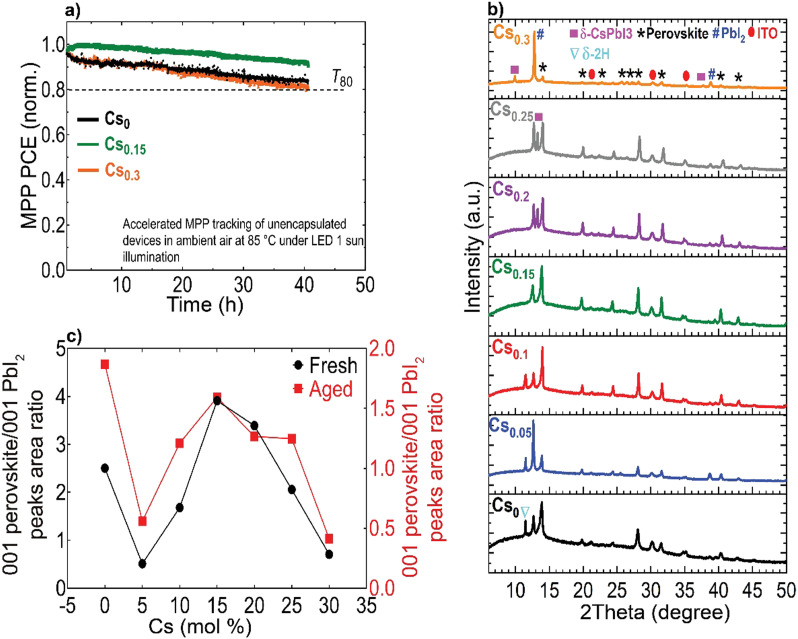
Accelerated aging tests and long-term structural stability: (a) MPP stability tracking (norm.) of unencapsulated perovskite devices under simulated sunlight at 85 °C in ambient conditions at a relative humidity level <20%, (b) XRD patterns of CsFA perovskite films, aged for 2280 hours under continuous simulated sunlight illumination at 35 °C in N_2_ atmosphere (the marked ‘*’ perovskite peaks include both the metrically cubic and superstructure phases) and (c) Relative intensities of 001 perovskite/001 PbI_2_ peaks for fresh and aged perovskite films.

Finally, we conducted accelerated ageing combining electrical and thermal stress testing under 1 sun illumination at a higher temperature of 85 °C in ambient atmosphere for unencapsulated devices at MPP conditions, following the standardized international summit on organic PV stability (ISOS) protocols^73^ (Fig. 6(a)). This accelerated ageing study uses the same device configuration shown in Fig. 5(a). The Cs_0.15_FA_0.85_PbI_3_ perovskite device maintained 95% of its peak performance for 40 hours. In contrast, the Cs_0_ and Cs_0.3_ PSCs degraded faster over the same measurement period with a similar ‘burn-in’ degradation period after 40 hours. To investigate the long-term intrinsic structural stability, we further performed XRD measurements for aged CsFA perovskite films that were stored in a N_2_ environment under full-spectrum sunlight illumination for a duration of 2, 280 hours (Fig. 6(b)). The structural analysis of the aged films reveals the emergence of the photo-inactive δ-2H FAPbI_3_ and γ-CsPbI_3_ phases manifested by Bragg peaks at 2Theta angles of 11.78° and 13.29°, respectively. The δ-2H FAPbI_3_ phase appears for the Cs_0_, Cs_0.05_ and Cs_0.1_ perovskite films, while the δ-CsPbI_3_ phase shows in Cs_0.2_ and Cs_0.25_ films. We point out that these phases were not detected in our fresh films (Fig. 2(a)). Significantly, the Cs_0.15_FA_0.85_PbI_3_ perovskite absorber retained the metrically cubic perovskite peaks with no detection of unwanted secondary phases, confirming the robust structural stability. These results validate our argument based on the TEM observations, that the presence of subtle structural imperfections such as hexagonal/orthorhombic phases, undetected with bulk XRD measurements, stimulate the growth of degradation sites under operational stressors, which deteriorate the device longevity. The behaviour of the relative intensities of the 001 perovskite to PbI_2_ peaks is similar for fresh and aged samples, as compared in Fig. 6(c), which demonstrates enhanced perovskite crystallinity for the Cs_0.15_FA_0.85_PbI_3_ perovskite absorber. We thus conclude that PbI_2_ particles, SFs and Cs-rich clusters are the most problematic phase impurities and are key drivers for absorber layer degradation on the long-term. Overall, our collective findings endow that despite the defect tolerance of perovskite materials with respect to the initial optoelectronic properties and device performance, these nanoscale defective phases seed degradation on the longer run which ultimately limit the device operational stability.

## Conclusion and outlook

Our work provides fundamental guidelines for engineering state of the art FA-rich perovskite absorbers pairing enhanced intrinsic stability with efficient device operation through the elimination of stacking faults in the desired α-FAPbI_3_ cubic phase at room temperature. We have studied the nanostructure-properties relationships in theCs_*x*_FA_1−*x*_PbI_3_ perovskites and assessed their impact on macroscopic devices with respect to performance and stability under operational conditions. We showed the effect of CsFA cation alloying on controlling the nanoscopic defects landscape and emphasized the vital role of attaining a minimal stacking fault perovskite absorber in enhancing the electronic conductivity, device performance and operational stability. Importantly, we urge for the significance of careful A-site compositional tuning in FA-rich perovskite absorbers that target high domain crystal quality. Our results represent a clear demonstration of linking the nanoscopic landscape to the device long-term operational stability, which attests to the importance of this fundamental investigation. Future research entailing the fundamental understanding of performance-limiting nanoscale phases in halide perovskites will emerge as a new domain for pushing the stability aspects in perovskite devices.

## Methods

### Materials

Lead-iodide (PbI_2_) beads (purity ≥99.999%) were purchased from Alfa Aesar. Anhydrous DMF (dimethylformamide), anhydrous DMSO (dimethyl sulfoxide), ethanol and ethyl-acetate (EA) were purchased from Sigma Aldrich. Formamidinium iodide (FAI) was purchased from Dyenamo. Cesium iodide (CsI) and MeO-2PACz were purchased from TCI. All chemicals were directly used as received.

### Perovskite thin film preparation

The perovskite composition used isCs_*x*_FA_1−*x*_PbI_3_. 1 M solutions of both neat FAPbI_3_ and CsPbI_3_ films were prepared in mixed solvents of DMF/DMSO (volume ratio 4 : 1) and then mixed by volume to get the desired nominal molar concentrations. Both precursor solutions contain a 10 mol% excess PbI_2_. A perovskite film with ∼350 nm thickness was then deposited on cleaned ITO glasses by spin coating 100 μl of the 1 M precursor solution (for PSC fabrication in a two-step spinning program: 1st at 1000 revolutions per minute (rpm), 200 rpm s^−1^ for 10 s and 2nd at 5000 rpm, 800 rpm s^−1^ for 35 s) or 0.6 M precursor solution to obtain a film thickness of ∼150 nm (for TEM specimen preparation in a one-step spinning program: 11 000 rpm, 11 000 rpm s^−1^ for 35 s). For the antisolvent quenching, 5 s before the end of the spin coating, 300 μl of EA was quickly dropped onto the perovskite surface. The deposited films were then immediately annealed at 150 °C for 30 min. All the film and device fabrication processes were done inside a glovebox filled with N_2_ atmosphere.

For TEM experiments: the perovskite films were directly spin-coated on ultra-thin C-coated Cu TEM grids that were sticked to ITO glass using a Kapton tape. To minimize any environmental potential-induced degradations such as moisture exposure, the samples were placed in a nitrogen (N_2_)-filled cylinder immediately after deposition inside the glovebox and then transferred into the TEM chamber in less than 5 mins of air exposure.

### PSCs fabrication

ITO glass substrates were washed with deionized water for 15 min followed by 10 min water-bath sonication. The cleaned substrates were treated by ultraviolet (UV) ozone for 10 min. After UV ozone treatment, 1 mg ml^−1^ of MeO-2PACz self-assembled monolayer (SAM) solution (hole-transport layer) dissolved in ethanol was spin-coated on substrates at 3000 rpm for 30 s in a N_2_-filled glovebox, followed by annealing at 100 °C for 10 min. The spin-coated CsFA perovskite films were deposited on top of MeO-2PACs and then annealed at 150 °C for 30 min. An electron-selective stack of C_60_ (20 nm, >99.95%, NanoC) was then thermally evaporated in a home-made evaporation system (base pressure <2 × 10^−6^ mbar, working pressure >3 × 10^−6^ mbar, evaporation rate of 0.3 Å s^−1^ as measured by quartz crystal monitors, aluminium oxide crucibles with power applied to the sources of ∼50 watt, the substrate holder was maintained at room temperature). A buffer layer of 25 nm of SnO_2_ was deposited by atomic layer deposition using an Oxford Instruments system at 100 °C using tetrakis(dimethylamino) tin and H_2_O as precursors. To finish the full-device, 130 nm of silver (Ag) was thermally evaporated through a shadow mask on the samples. During all the thermal evaporations, the deposition rate was first stabilized to the targeted value before opening the substrate shutter. We note here that the TEM specimens were exposed to only 4 mins of UV-ozone treatment.

### TEM characterization

BF micrographs and SAED patterns were acquired on a Talos F200S TEM operating at 200 kV and located in the Centre interdisciplinaire de Microscopie électronique (Cime) of EPFL, Lausanne campus. To minimize any possible electron beam-induced artifacts, we used low-dose TEM imaging conditions, with an electron dose rate of ∼1 e Å^−2^ s^−1^. All the TEM BF micrographs and diffraction patterns were recorded from previously unexposed regions of the sample. Furthermore, the specimen was never tilted and the SAED patterns were taken at whatever orientation the domains were found in. A small objective aperture of 10 μm size was used to enable the record of localised DPs. The SAED patterns were analysed using JEMs software.

### (Temperature dependent) ultra-sensitive EQE

A UV/VIS/NIR spectrophotometer (PerkinElmer, Lambda 950) was used as a source of monochromatic light. The probe light was physically chopped at 273 Hz and directed with different optical components onto the device under test (DUT). Prior recording the DUT response with a lock-in amplifier (Stanford Research, SR860), the DUT photocurrent was amplified by a current pre-amplifier (FEMTO, DLPCA-200). The DUT was mounted in an electrically shielded sample holder (Linkam, LTS420E-P); for temperature dependent measurements, the sample holder was connected to a liquid nitrogen pump (Linkam, LNP96) and a temperature controller (Linkam, T96). For calibration, a NIST-calibrated Si (Newport, 818-UV) and Ge (818-IR) photodiode sensor were used as reference devices. Detailed information about the EQE apparatus is provided elsewhere.^46^

### Potentiostatic polarization measurements

Gold contacts with a thickness of 100 nm were thermally evaporated (Nexdep, Angstrom Engineering) onto the perovskite films through a shadow mask with a diameter of 1 mm to measure the electronic and ionic conductivity. The complex impedance was measured from 0.1 Hz to 1 MHz with an amplitude of 50 mV using a Paios measurement system (Fluxim AG). The complex electric modulus was fitted to extract the effective ionic conductivities. DC polarisation curves were also acquired with the Paios instrument by applying a constant potential of 0.2 V and measuring the current over 10 minutes. The electronic conductivity was calculated from the steady-state current value.

### XRD

Carried out in an Empyrean diffractometer (Panalytical) equipped with a PIXcel-1D detector, located at EPFL, Lausanne campus. The XRD patterns were obtained using the Cu Kα radiation (wavelength of 1.54 Å).

### SEM

SEM images were acquired with an acceleration voltage of 3 kV and probe current of 110 pA using an in-lens detector in a Zeiss GeminiSEM 450 microscope. The dwell time for image acquisition was 10 μs per probed pixel.

### AFM

Performed using an AFM Dimension Edge Bruker microscope with an aluminium (Al)-coated Si tip (Model TESPA-V2) in tapping mode at ambient atmospheric conditions. Scans were performed over 2 μm at 512 pixels and 1 Hz frequency.

### KPFM

KPFM maps were generated using an AFM Dimension Edge Bruker microscope with a Pt-coated Si tip (Model SCM-PIT-V2), having a spring constant (*k*) = 3 N m^−1^. Scans were performed over 2 μm at 512 pixels and 1 Hz frequency in a dual-pass setup, the first pass to record topography and the second to measure the contact-potential difference (CPD) with a tip potential of 5 V (both passes are in tapping mode). The WF of the Pt tip was precisely calibrated by measuring the CPD relative to a freshly cleaved highly oriented pyrolytic graphite (HOPG); a material with a standard work function of about 4.6 eV. This allowed us to estimate locally the surface potential values of the different perovskite films with high spatial resolution. Each sample was scanned in at least three random locations to ensure reliable measurements (ESI,† Fig. S9e). The measurements were done in ambient atmosphere.

### SIMS

To investigate the elemental distributions, high resolution SIMS imaging was performed using a 25 keV Ne^+^ primary ion beam. SIMS imaging was performed in helium/neon ion microscope (Zeiss NanoFab, Peabody, USA) coupled with a magnetic sector mass spectrometer developed at the Luxembourg Institute of Science and Technology.^74^ The samples were biased to +500 V or −500 V for the acquisition of positive or negative secondary ions respectively. The secondary ions that were detected from the perovskite samples are ^208^Pb^+^, ^133^Cs^+^, ^12^C^14^N^−^ and ^127^I^−^. The images were acquired as a matrix of 512 × 512 pixels with a 1 ms dwell time. The raster size was 12 × 12 μm^2^. Note that the region of interest for the SIMS images for positive polarity is different from that for negative polarity, as well as secondary electron (SE) images.

### C-AFM

Photo-current maps were acquired using an AFM Dimension Edge Bruker microscope with a Pt-coated Si tip (Model SCM-PIT-V2), having a spring constant (*k*) = 3 N m^−1^. Scans were performed over 2 μm at 512 pixels and 1 Hz frequency in a dual-pass setup, the first pass in tapping mode to measure topography while the second in contact mode at *J*_SC_ conditions to extract photo-current. Similar to AFM and KPFM, the measurements were done in ambient atmosphere.

### GIWAXS

Thin-film diffraction data were collected at the BM01 end station of the Swiss-Norwegian Beamlines at the ESRF (Grenoble, France), on the Pilatus@SNBL diffractometer.^75^ The data were recorded with Pilatus3 2M detector at a grazing incidence of 0.4°, with wavelength of 0.64 Å. The data processing and inspection of the powder diffraction profiles were done with BM01 software tools such as BUBBLE^75^ and MEDVED.^76^

### TRMC

The TRMC technique was used to investigate both the mobilities and lifetimes of photo-generated charge carriers in semiconductor materials. With this technique, the reduction in microwave power (Δ*P*(*t*)/*P*) induced by a laser pulse (repetition rate 10 Hz) was related to the change in conductance (Δ*G*(*t*)) by the sensitivity factor *K*:1
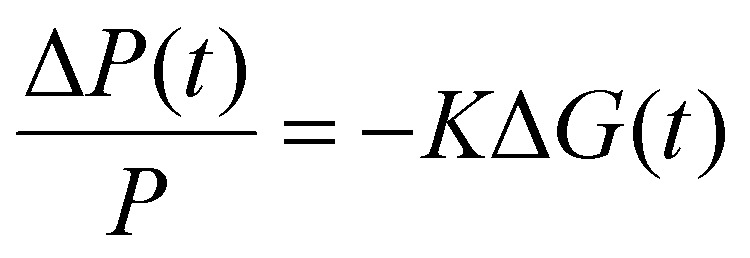


The TRMC signal is expressed in the product of mobility (*μ*_e_ + *μ*_h_) and charge carrier yield *φ*, which was calculated from the maximum change in photoconductance Δ*G*_max_:2
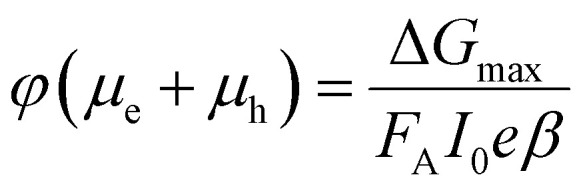
Where *F*_A_ is the fraction of light absorbed by the sample at the excitation wavelength, *I*_0_ is the laser intensity in number of photons per unit area per pulse, *e* is the elementary charge and *β* is the ratio of the inner dimensions of the microwave cell. The samples were placed in a sealed microwave cavity cell inside the glovebox to ensure that they are not exposed to ambient conditions during the measurement.

### PLQY

Light from a laser diode (excitation wavelength = 523 nm) is coupled into a fibre directed into the integrating sphere and then, sample is illuminated. The emission from the sample is homogenized by multiple reflections within the integrating sphere and coupled out into another fibre that is connected to a spectrometer.

### Device performance

In-house current–voltage (*JV*) measurements were obtained on a temperature-controlled vacuum chuck at 25 °C, if not stated otherwise, using a two-lamp (halogen and xenon) class AAA WACOM sun simulator with an AM1.5 G irradiance spectrum at 1000 W m^−2^. Shadow masks were used to define the illuminated area (here 0.1 cm^2^). The cells were measured with a scan rate of 100 mV s^−1^ (using an integration time of 0.02 s and a delay of 0.02 s for each data point).

### Stability experiments

Accelerated ageing measurements for 40 h were performed with the advanced stability platform Litos by Fluxim AG, Switzerland. The samples were kept at 85 °C, illuminated by white LEDs at 1 sun-equivalent light intensity, and 12 pixels were kept at MPP. Standard stability tests were conducted in a customized environmental chamber (Cicci Research s.r.l.) under continuous nitrogen flow and at 35 °C for over 2000 h. Illumination was provided by an LED solar simulator calibrated to approximate AM1.5G, and the devices were at open-circuit condition.

## Author contributions

M. O., C. M. W. and A. H. W. conceived and designed the work. M. O. produced the original draft and all co-authors contributed to editing the manuscript. C. M. W., Q. J., A. H. W. and C. B. contributed to the investigation, methodology and analysis of the work. M. O. prepared the TEM specimens and collected the data. M. O., Q. J. and A. H. W. interpreted and analysed the SAED data. S. Z. and A.A. performed ultra-sensitive EQE measurements. S. T., S. E. and T. W. conducted and analysed the SIMS experiments. M. O. performed the AFM, KPFM and C-AFM experiments. M. O., C. M. W., D. A. J. and C. B. contributed to the planning and analysis of the AFM-analytical data. M. H. F. performed the potentiostatic polarization measurements. D. C. assisted in the analysis of the GIWAXS data. M. O. carried out the XRD experiments. M. O. fabricated the perovskite devices and carried out the stability tests. J. Z. and T. S. performed the TRMC measurements and provided the data analysis. C. M. W. and D. A. J. helped in analysing the spectroscopy data. A. J. and A. K. contributed to the analysis of the work. S. J., S. Z. and B. R. assisted in performing the accelerated device aging tests and contributed to the analysis of the work.

## Conflicts of interest

The authors declare no conflicts of interest.

## Supplementary Material

EE-017-D4EE00901K-s001
