# Supplementary material for: Alleviating nanostructural phase impurities enhances the optoelectronic properties, device performance and stability of cesium-formamidinium metal–halide perovskites
Source: Energy Environ Sci. 2024 May 9;17(11):3832–47. doi: 10.1039/d4ee00901k (PMC11149396; doi:10.1039/d4ee00901k)
Supplement: EE-017-D4EE00901K-s001 [file EE-017-D4EE00901K-s001.pdf]

Electronic Supplementary Material (ESI) for Energy & Environmental Science.
This journal is © The Royal Society of Chemistry 2024

Alleviating Nanostructural Phase Impurities Enhances the Optoelectronic Properties, Device Performance and Stability of Cesium-Formamidinium Metal-Halide Perovskites

Mostafa Othman*, Quentin Jeangros, Daniel A. Jacobs, Moritz H. Futscher, Stefan Zeiske, Ardalan Armin, Anaël Jaffrès, Austin G. Kuba, Dmitry Chernyshov, Sandra Jenatsch, Simon Züfle, Beat Ruhstaller, Saba Tabean, Tom Wirtz, Santhana Eswara, Jiashang Zhao, Tom Savenije, Christophe Ballif, Christian M. Wolff*, Aïcha Hessler-Wyser*

Supplementary information Note1. The quantification of the $\{111\}_C$ SFs and δ-$CsPbI_3$ domains is based on counting the diffracting domains, that solely appear with a dark black contrast in the field of view of the BF micrographs. As halide-perovskites degrade quickly under the electron beam, the specimen was never tilted to change the diffraction contrast and therefore any BF image was recorded as initially viewed. Furthermore, any domain that appears to be grey (not in full diffraction conditions) was never counted in our analysis of the $\{111\}_C$ SFs and δ-$CsPbI_3$ domains. Within the field of view of each micrograph for all samples, any diffracting domain having the characteristic striped contrast was counted as the $\{111\}_C$ SFs while the δ-$CsPbI_3$ phase is based on the number of indexed domains that are found to be in this phase. According to this methodology, no SFs were found in all the $[011]_C$ zone axes of the cubic phases of $Cs_{0.15}FA_{0.85}PbI_3$ samples among multiple experiments. However, for the sake of clarity and precision, we cannot exclude the probability about the presence of a random SF domain that we were not able to identify/index as it was not in diffraction conditions or within our field of view.

Supplementary information Note2. The ionic conductivity is obtained by impedance spectroscopy measurements of ITO/Perovskite/Cu. The conductivity between two parallel plates is defined as

$$\sigma = \frac{1d}{RA}$$

where R is the resistance, d is the thickness of the layer, and A is the area. The geometric capacity is given by

$$C = \frac{\varepsilon\varepsilon_0 A}{d}$$

where ε is the material's dielectric constant and ε_0 the vacuum permittivity. Together with the RC time constant, the conductivity can be expressed as

$$\sigma_{ion} = \omega_{max}\varepsilon\varepsilon_0$$

where ω_{max} is the frequency at which migration occurs. The electric modulus was used to determine ω_{max} and ε, as shown in **Fig. 1a**. The advantage of the electric modulus is that it suppresses signals with low impedance, such as interface polarization, that can overlay effects caused by long-range conduction (see **Fig. 1b**)[1].

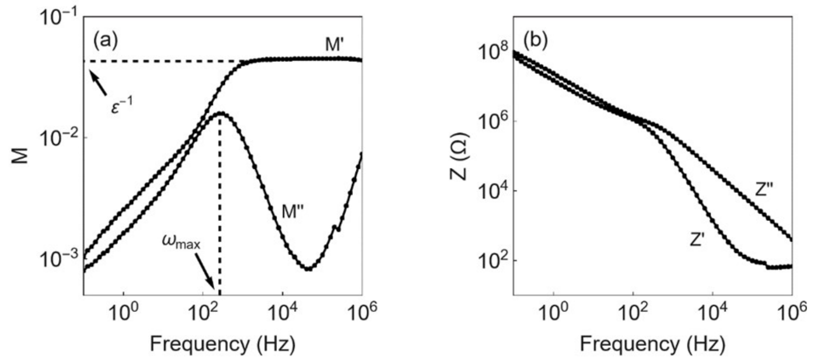

Figure 1. a) The real and imaginary part of the modulus and b) the impedance of $Cs_{0.05}FA_{0.95}PbI_3$ measured by impedance spectroscopy. At low frequencies, interfacial polarization due to the accumulation of ions at the interfaces dominates the impedance, which is suppressed in the modulus. ω_{max} and ε are obtained by the imaginary and the real part of the modulus, respectively.

The electronic conductivity is obtained by current transient measurements of ITO/Perovskite/Au. The steady-state current I after applying a voltage bias U is used to calculate the electronic conductivity with

$$\sigma_{eon} = \frac{I\,d}{UA}.$$

An exemplary current transient is shown in **Fig. 2a**. After applying a voltage bias, a current transient is observed due to mobile ions within the perovskite drifting toward the interfaces. After a few hundred seconds, all the mobile ions have moved, and the current can be attributed to the electronic conductivity. To avoid internal electric field contributions, for example, due to the different work functions of the electrodes, we took the average current measured after applying a positive and a negative voltage bias, i.e., $I = (I_{+U} + I_{-U})/2$. As a bias, we use 0.2 V, which is in the approximate linear regime of the JV curve.

The concentration of mobile ions is estimated by integrating the current response after applying a bias, subtracting the contribution by electronic conductivity. With increasing bias, the concentration of mobile ions increases until it levels off close to 0.8 V (**Fig. 2b**). Note that we limit the bias to 0.8 V as higher biases sometimes irreversibly degrade the device. As a result, the obtained mobile ion density corresponds to a lower bound of mobile ions. We find that the concentration of mobile ions extracted at a bias of 0.8 V remains approximately constant with increasing Cs content (**Supplementary Fig. 12**). This indicates that the ion conductivity change observed in impedance spectroscopy is related to a change in the mobility of mobile ions.

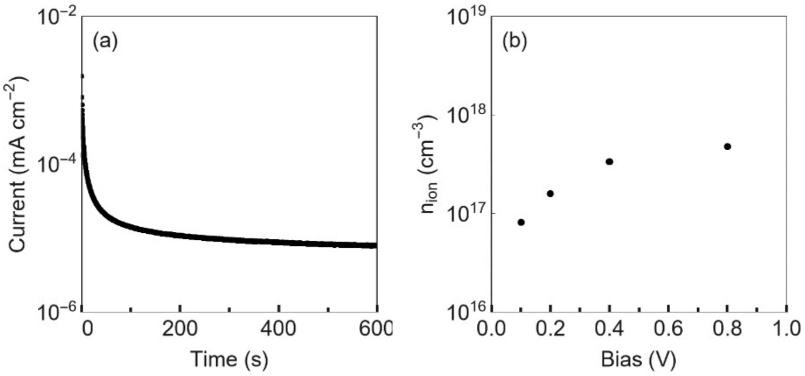

Figure 2. a) Current transient of $Cs_{0.05}FA_{0.95}PbI_3$ measured after applying a bias of 0.2 V. The initial decay is related to the redistribution of mobile ions within the perovskite. The steady-

state current at long times can be attributed to electronic conductivity and b) The concentration of mobile ions as a function of applied bias for $Cs_{0.05}FA_{0.95}PbI_3$

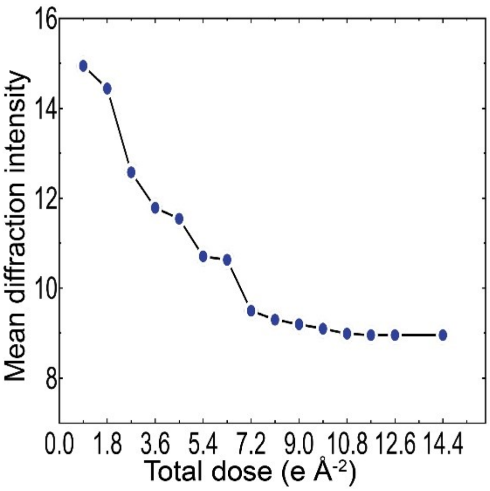

Supplementary Figure S1. Beam-damage analysis of atomic-lattice planes loss as a function of total accumulated dose for a twinned reflection in a cubic $FAPbI_3$ phase near the $[011]_C$ zone axis.

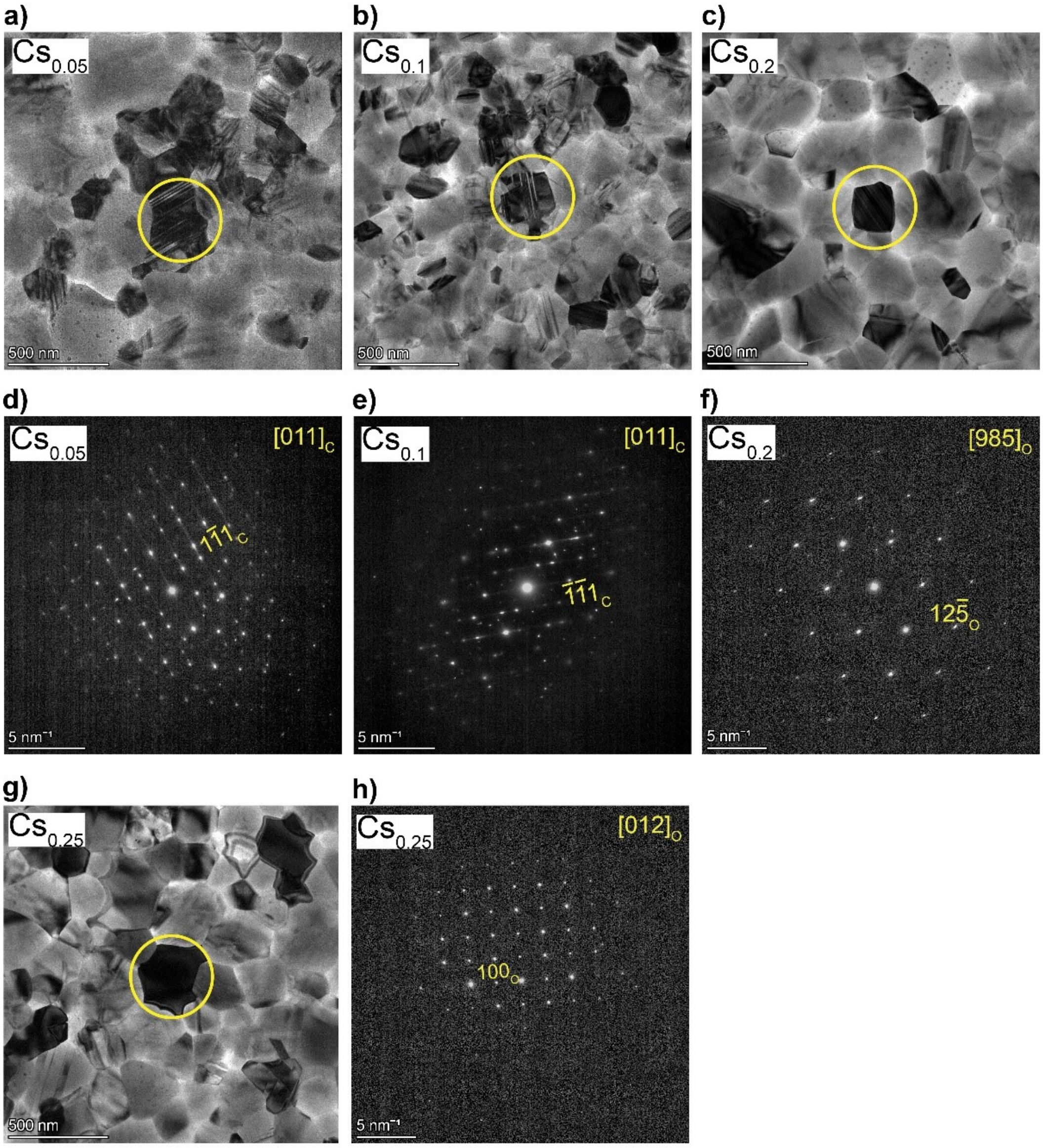

Supplementary Figure S2. a, b & d and e) BF TEM images and associated SAED patterns of cubic $FAPbI_3$ phase oriented near the $[011]_c$ zone axis for $Cs_{0.05}FA_{0.95}PbI_3$ and $Cs_{0.1}FA_{0.9}PbI_3$ perovskites, respectively, c, f & g and h) BF TEM images and SAED patterns of $Cs_{0.2}FA_{0.8}PbI_3$ and $Cs_{0.25}FA_{0.75}PbI_3$ perovskite thin films that could be indexed to an orthorhombic $CsPbI_3$ phase oriented near the $[985]_O$ and $[012]_O$ zones axes, respectively. The "yellow" circles represent the placed aperture on specific domains taking locally the SAED patterns.

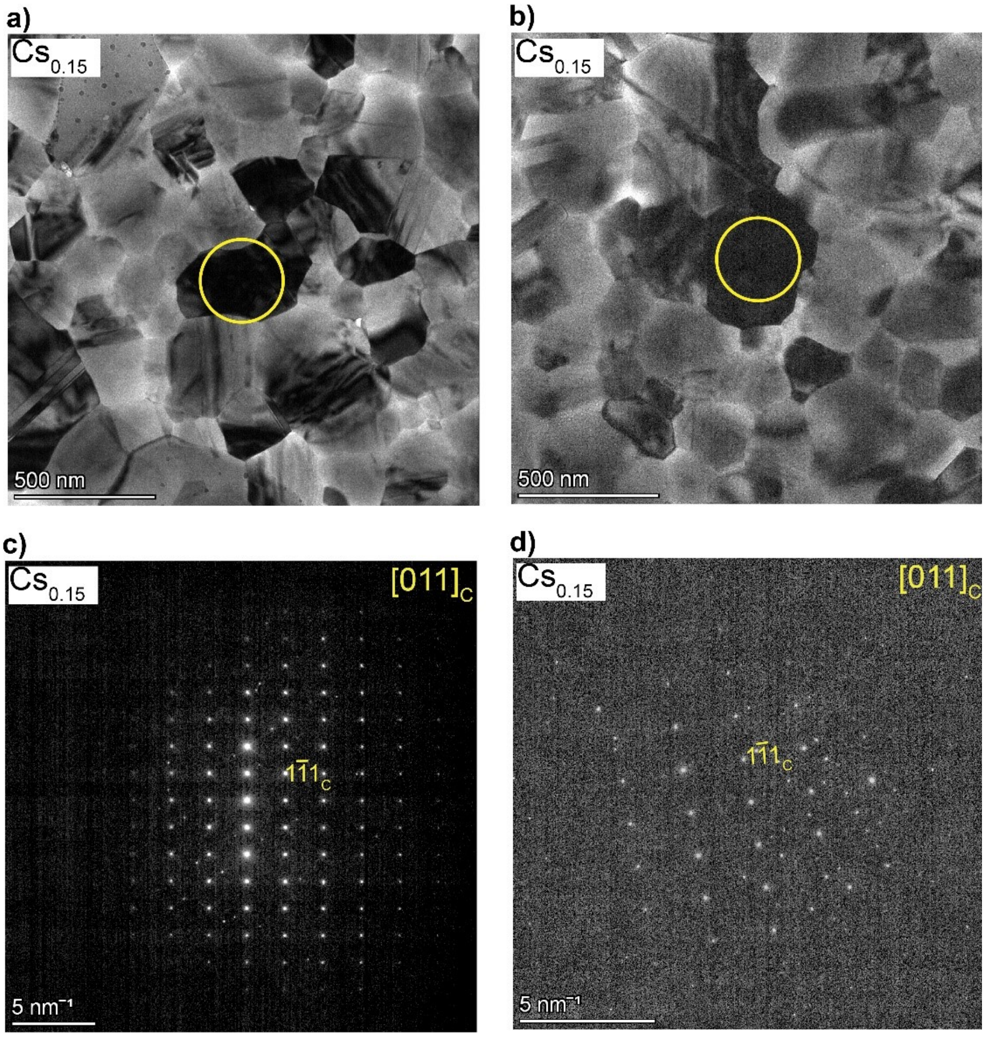

Supplementary Figure S3. a, b & c, d) TEM BF images of a $Cs_{0.15}FA_{0.85}PbI_3$ thin film absorber and corresponding SAED patterns of cubic $FAPbI_3$ phases oriented near the $[011]_C$ zone axis. The "yellow" circles represent the placed aperture on specific domains to take local DPs.

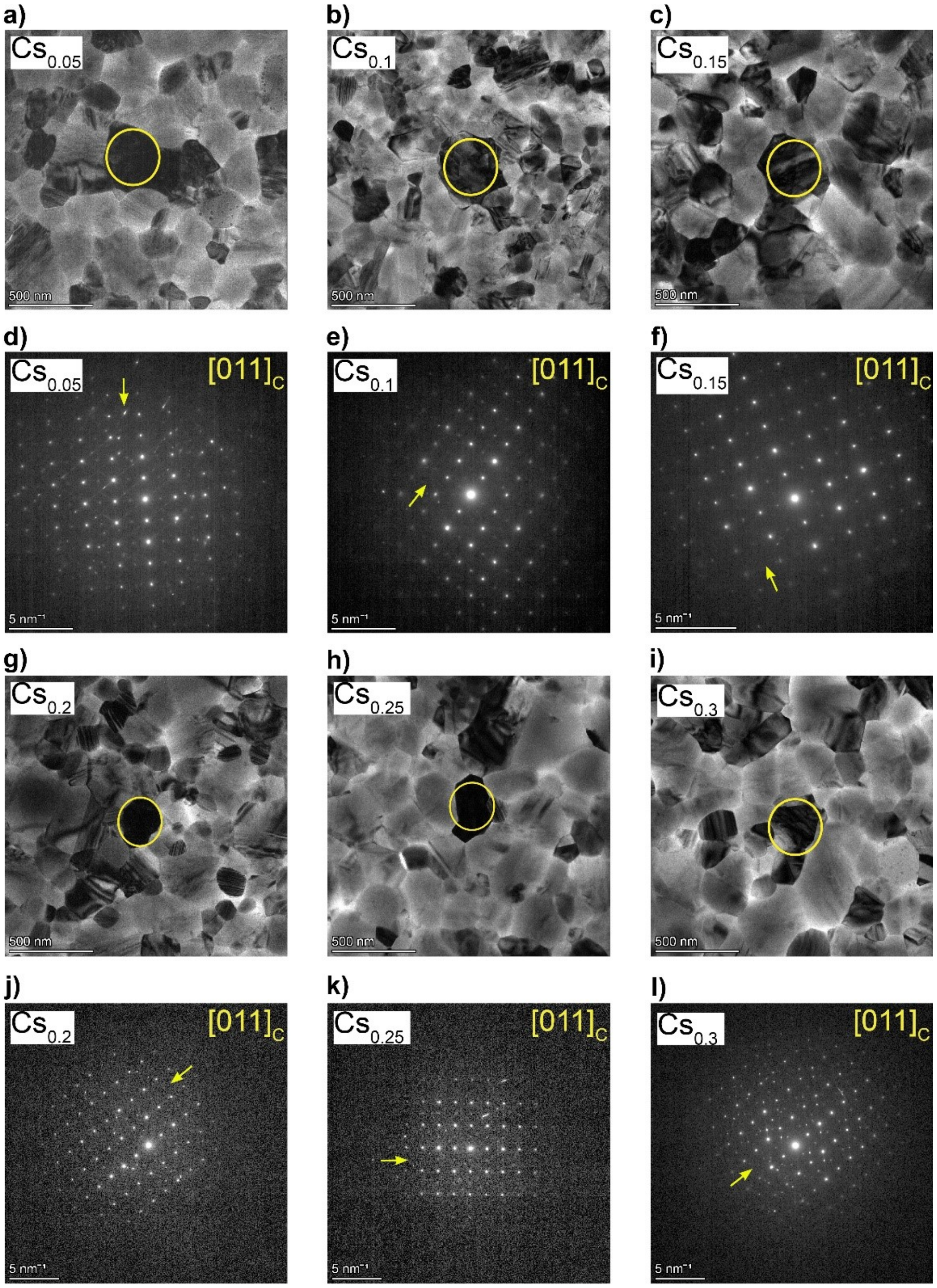

Supplementary Figure S4. a-i) TEM BF images and corresponding SAED patterns for $Cs_xFA_{1-x}PbI_3$ perovskite thin films that are indexed to a cubic superstructure $FAPbI_3$ phase oriented near $[011]_C$ zone axis. The "yellow" arrows highlight kinematically forbidden reflections to occur in a cubic structure. The "yellow" circles represent the placed aperture on specific domains taking locally the SAED patterns.

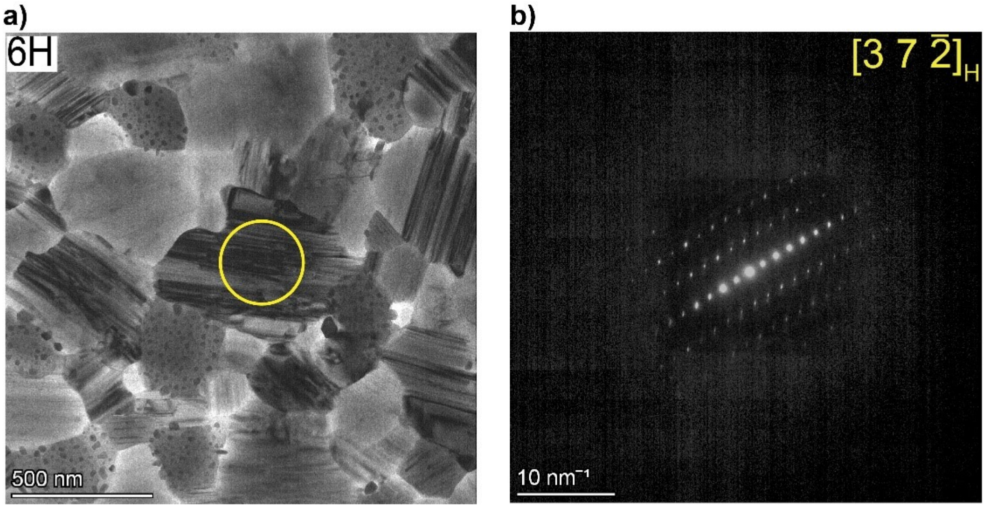

Supplementary Figure S5. *In situ* heating of a $FAPbI_3$ thin film in TEM at 170 °C: a) BF TEM image of the heated film b) SAED pattern corresponding to the domain encircled in "yellow" that is indexed to a 6H-$FAPbI_3$ phase oriented along the $[37\bar{2}]_H$ zone axis. The black dots shown across the film surface are indicative of PbI_2 particles generated as a by-product of the perovskite film decomposition.

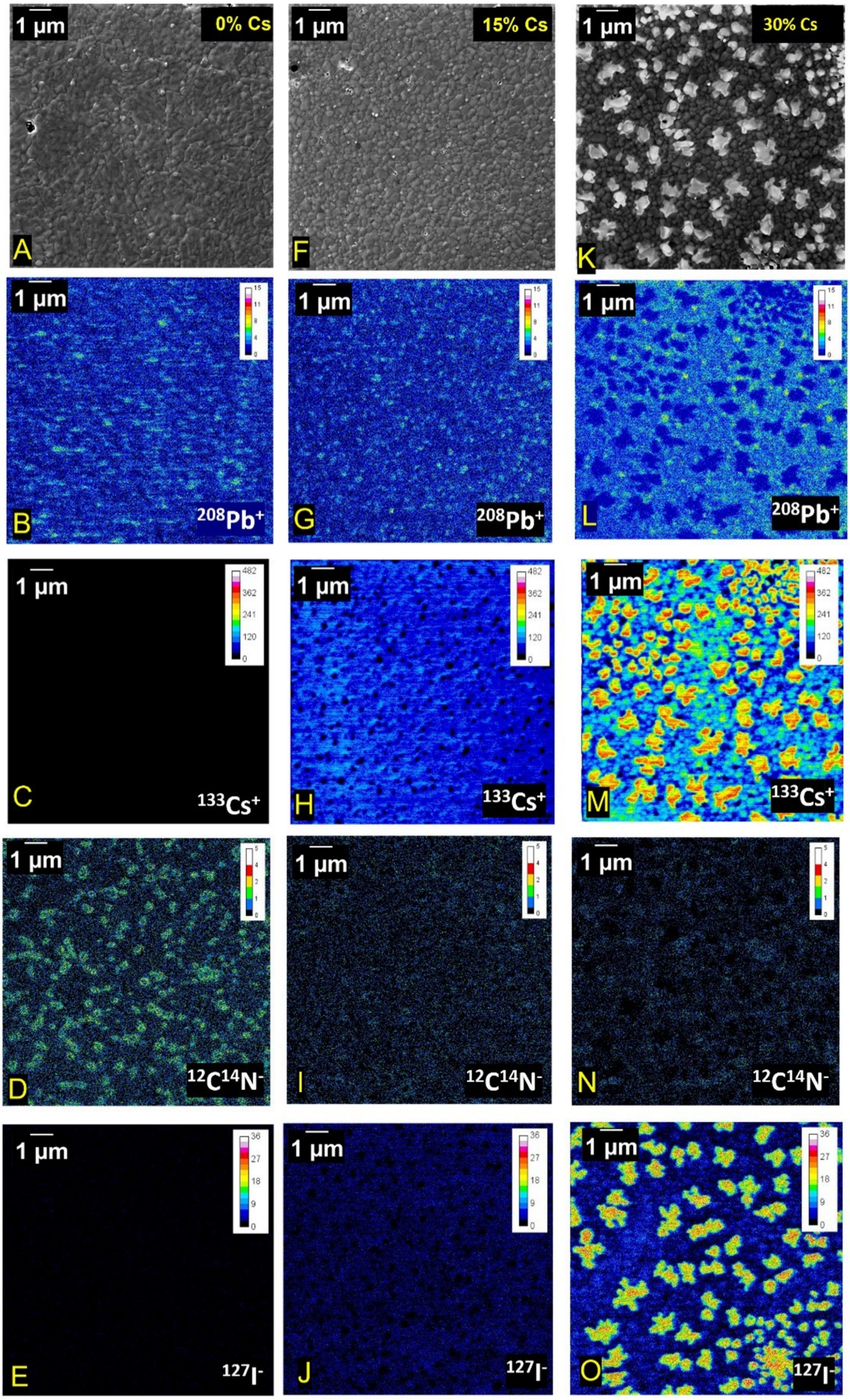
1 µm
0% Cs
15% Cs
30% Cs
A
F
K
B
G
L
$^{208}Pb^{+}$
C
H
M
$^{133}Cs^{+}$
D
I
N
$^{12}C^{14}N^{-}$
E
J
O
$^{127}I^{-}$

Supplementary Figure S6. Secondary electron images of a) $FAPbI_3$, f) $Cs_{0.15}FA_{0.85}PbI_3$, k) $Cs_{0.30}FA_{0.70}PbI_3$ perovskite films and SIMS images of the b-e) $FAPbI_3$, g-j) $Cs_{0.15}FA_{0.85}PbI_3$, l-o) $Cs_{0.30}FA_{0.70}PbI_3$ films.

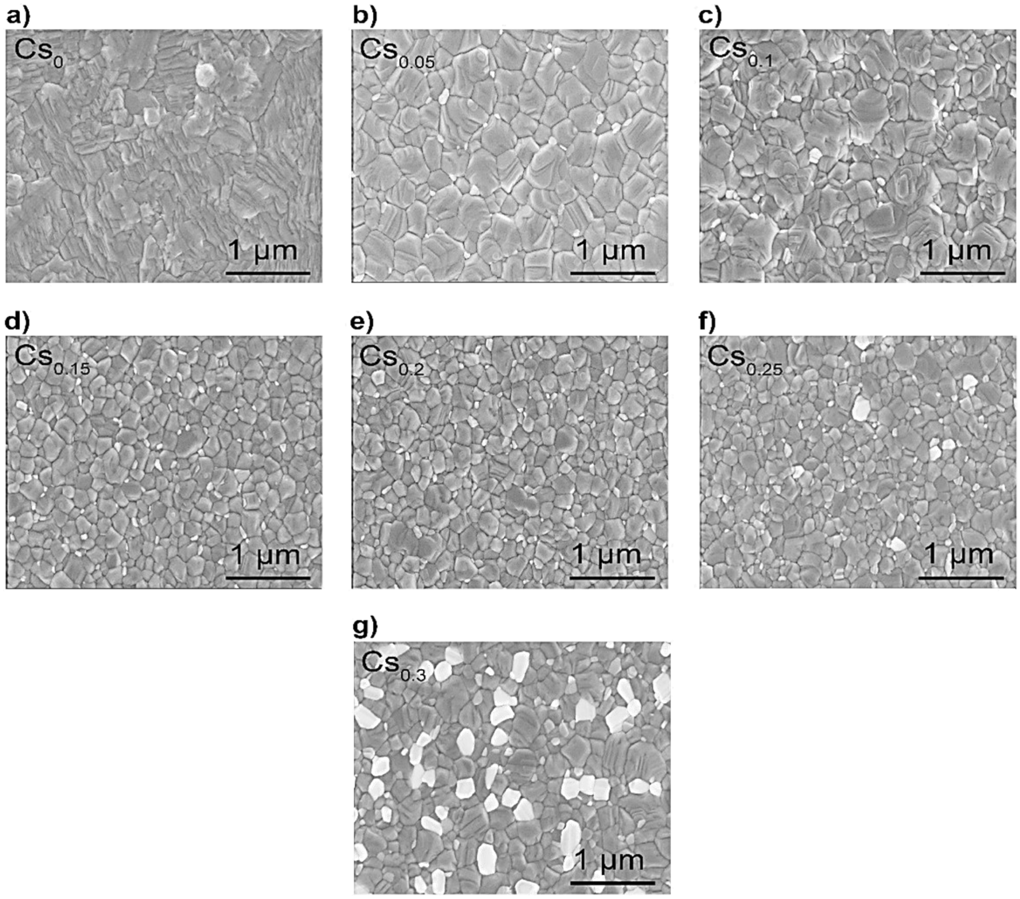

Supplementary Figure S7. a-g) Top-view SEM morphology micrographs of the $Cs_xFA_{1-x}PbI_3$ perovskite films.

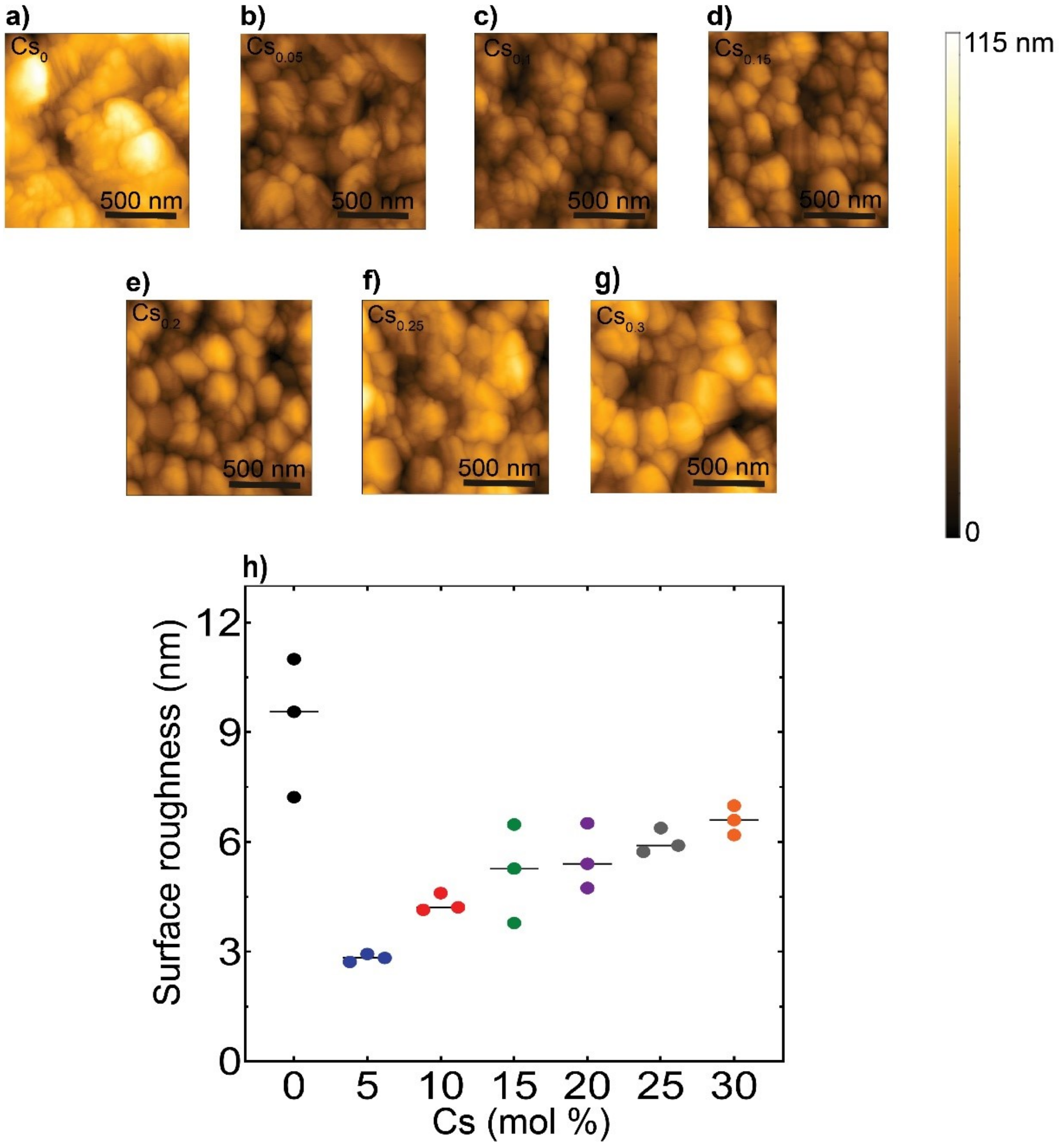

Supplementary Figure S8. a-g) AFM images of the CsFA perovskite films and h) Statistical analysis of the root mean square (rms) roughness values as a function of Cs content. The three dot points represent surface roughness values extracted from three different scan areas.

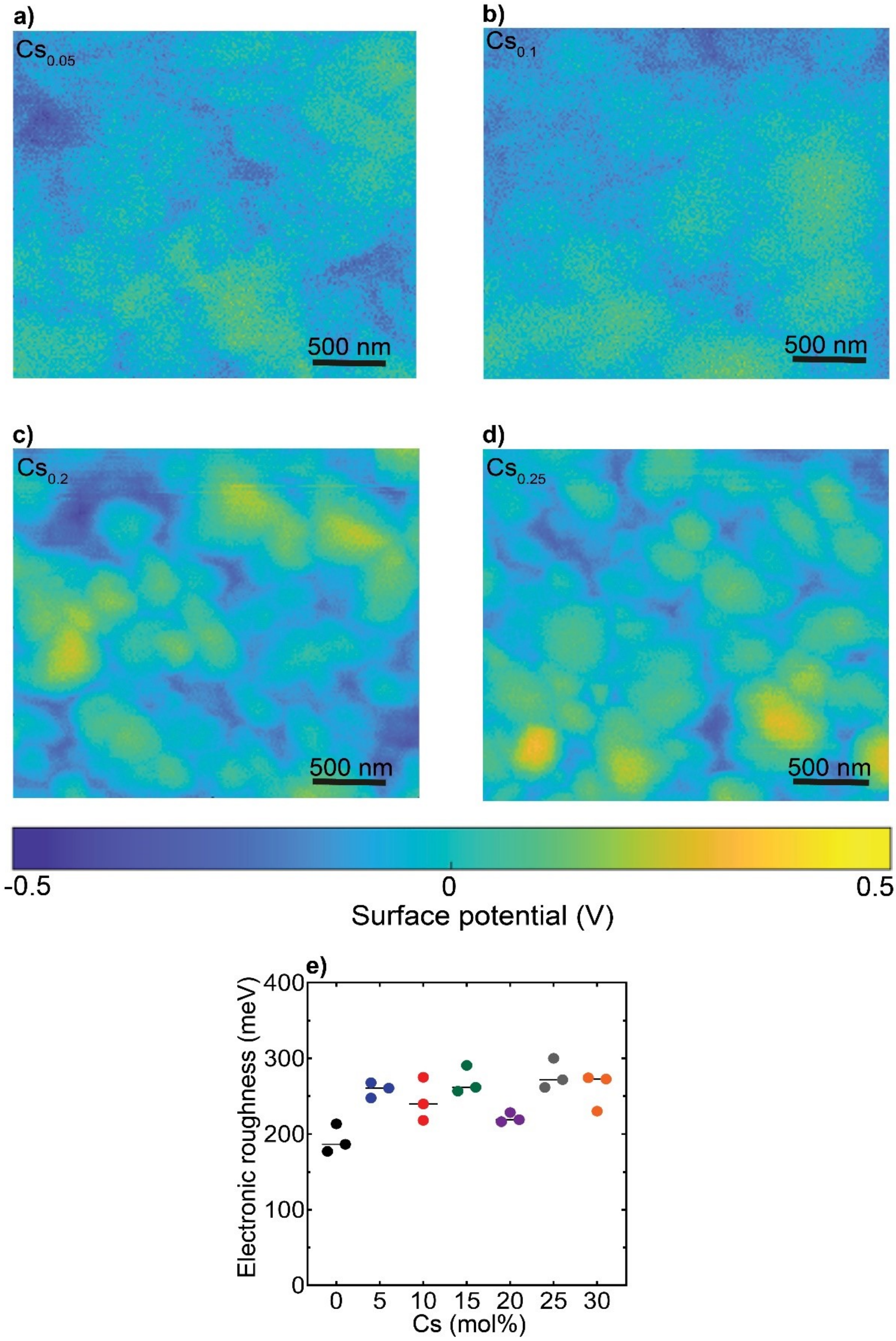

Supplementary Figure S9. a-d) KPFM maps of CsFA perovskite films and e) Dot-points analysis representing surface potential variations and taken from different scan areas to ensure measurements reliability.

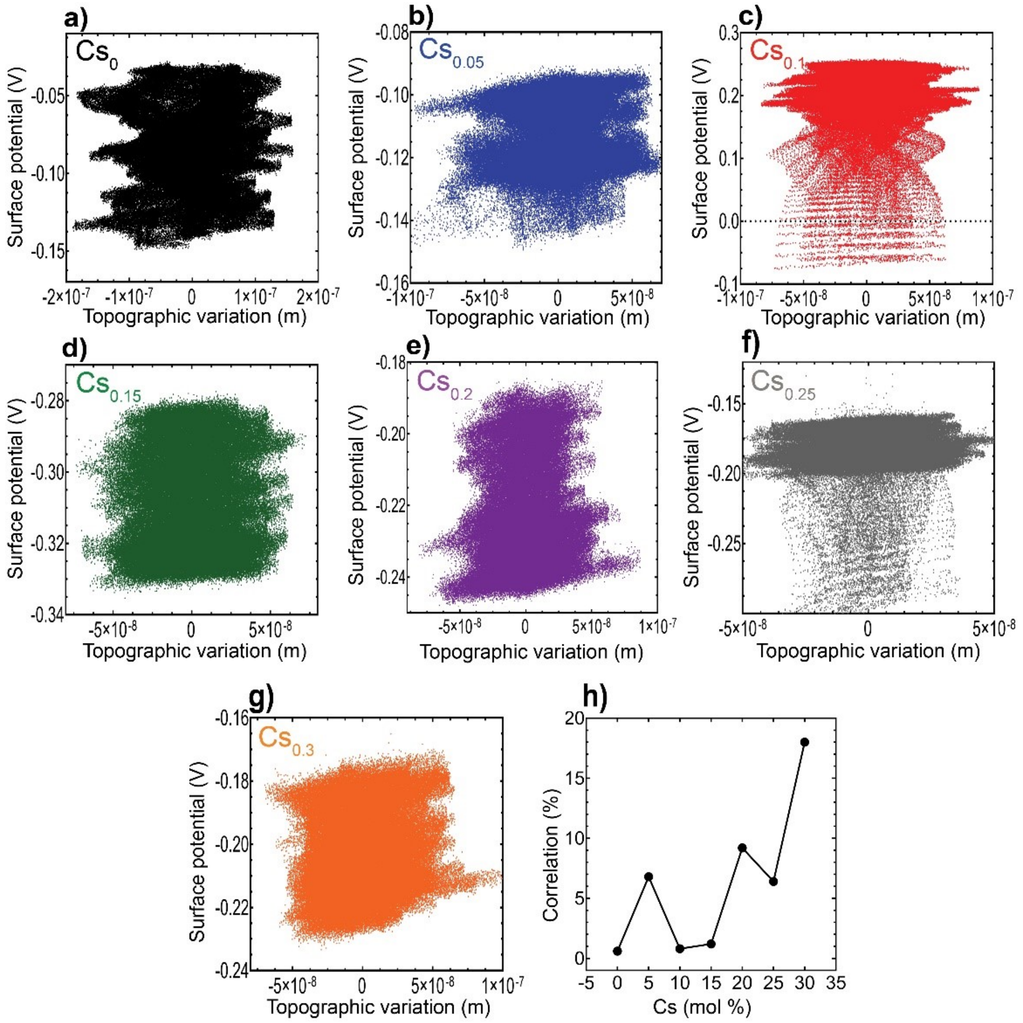

Supplementary Figure S10. a-g) Correlation maps between the surface topography and potential values for the different CsFA perovskite films and h) Summary plot for the obtained correlation values as a function of the Cs molar content.

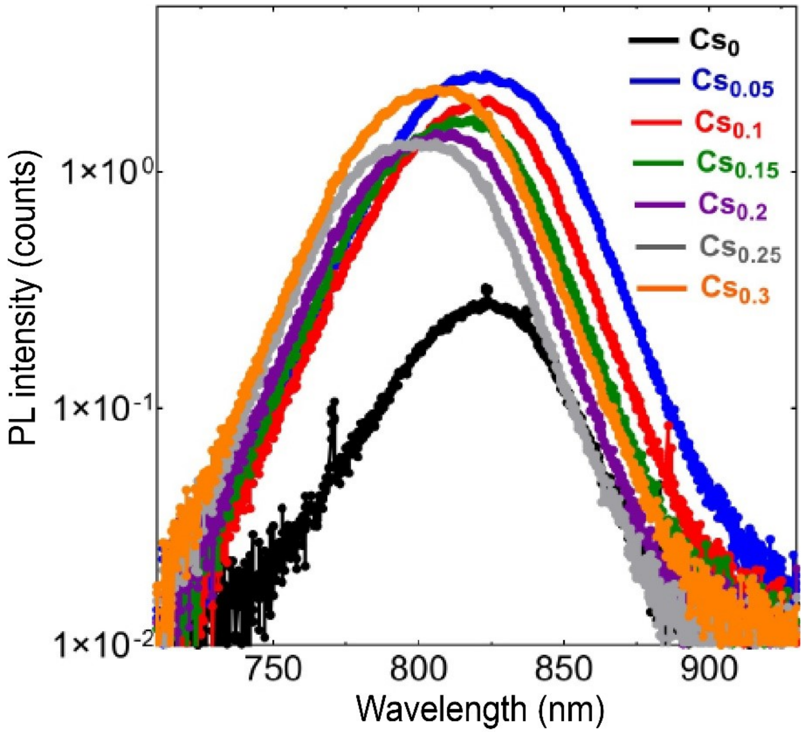

Supplementary Figure S11. PLQY of the $Cs_xFA_{1-x}PbI_3$ perovskite films.

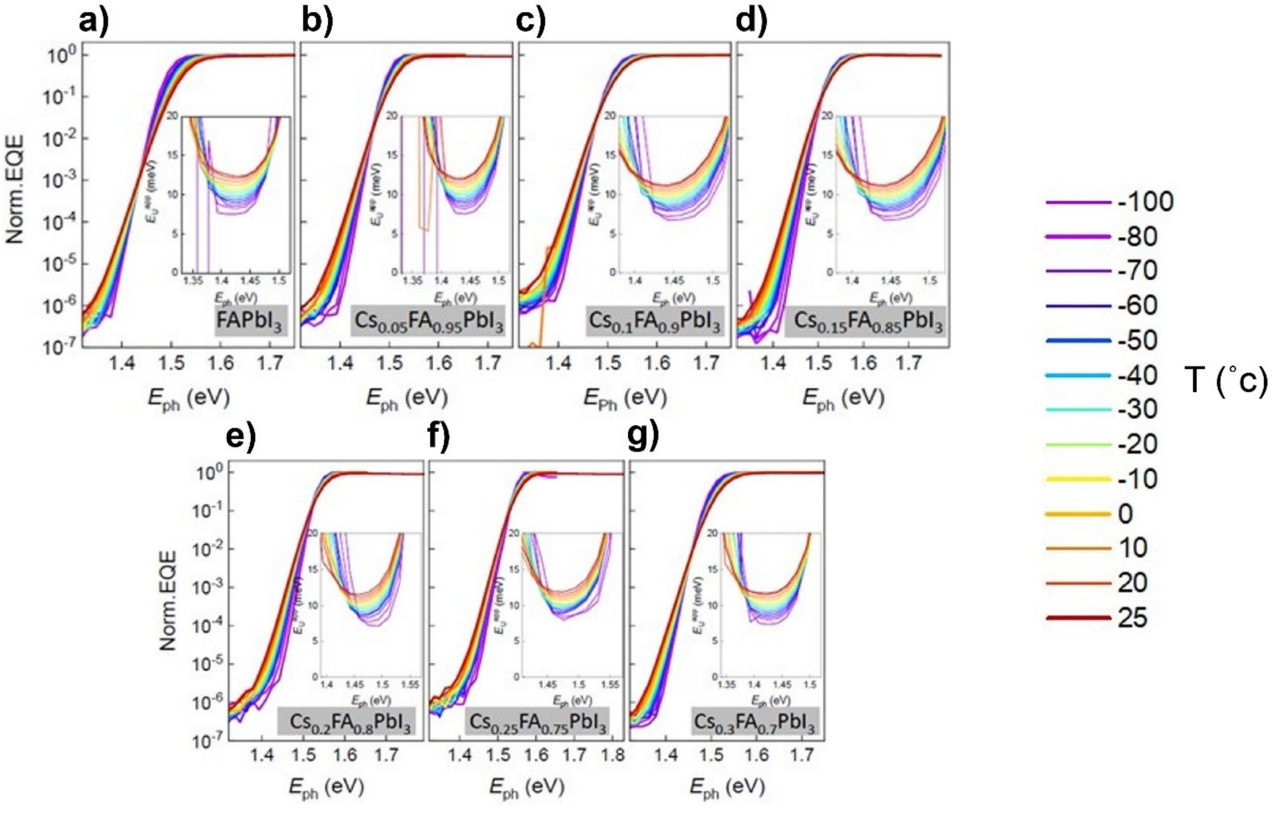

Supplementary Figure S12. a-g) Normalized external quantum efficiency (EQE) of $Cs_xFA_{1-x}PbI_3$ perovskite devices plotted as a function of photon energy and compared at different

temperatures. The inset shows the calculated apparent Urbach energy spectrum from which the Urbach energy was determined.

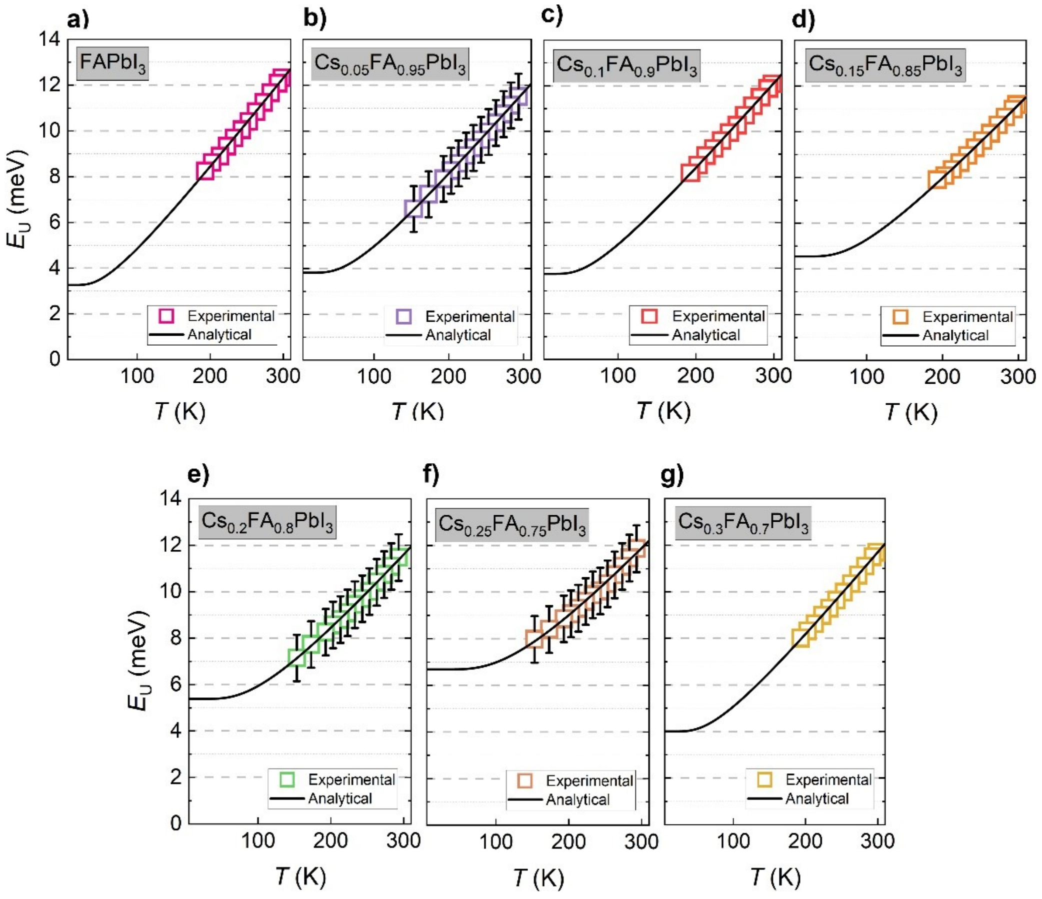

Supplementary Figure S13. e-g) Experimentally obtained (symbols) Urbach energy plotted as a function of temperature and compared for (from left to right) different $Cs_xFA_{1-x}PbI_3$ perovskite devices. Solid lines are fits to the Einstein solid model. The corresponding fit parameters are provided in Table 1.

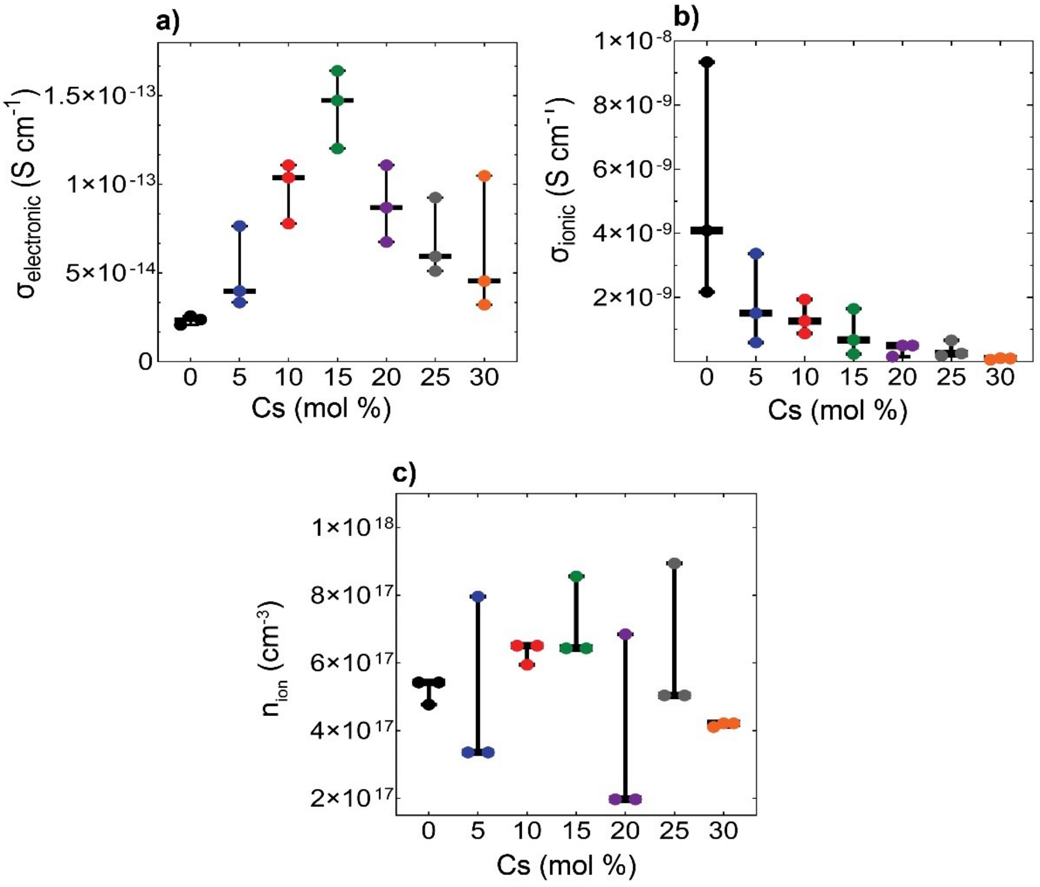

Supplementary Figure S14. Potentiostatic polarization measurements for the CsFA perovskite thin films: a) electronic conductivity, b) ionic conductivity and c) ion-densities. The "three-dot" points represent multiple measurements taken per sample.

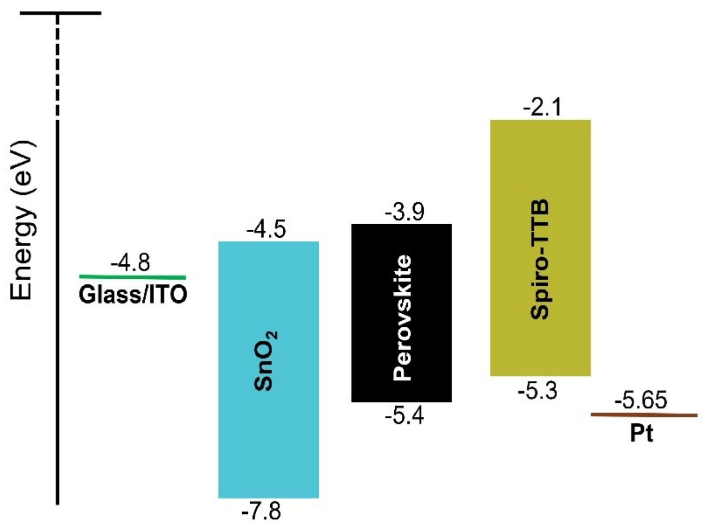

Supplementary Figure S15. Energy band diagram for the different layers and the Pt tip.

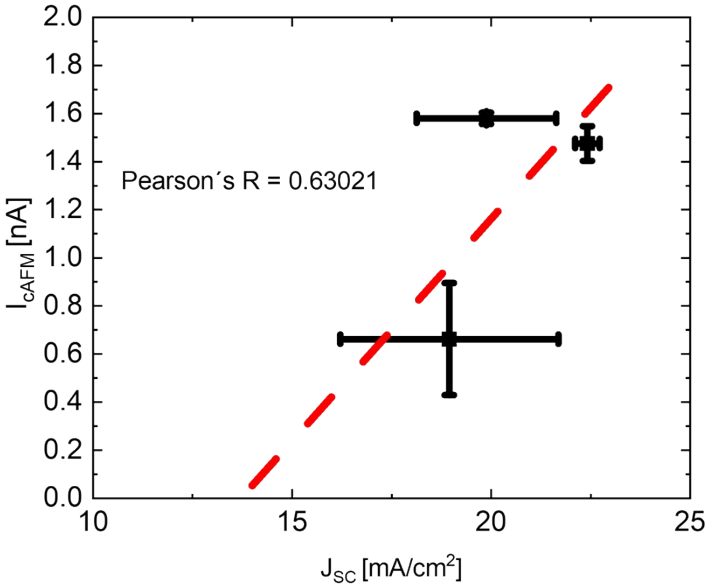

Supplementary Figure S16. Correlation figure between J_{SC} and I_{cAFM} values obtained from full and pseudo-devices, respectively.

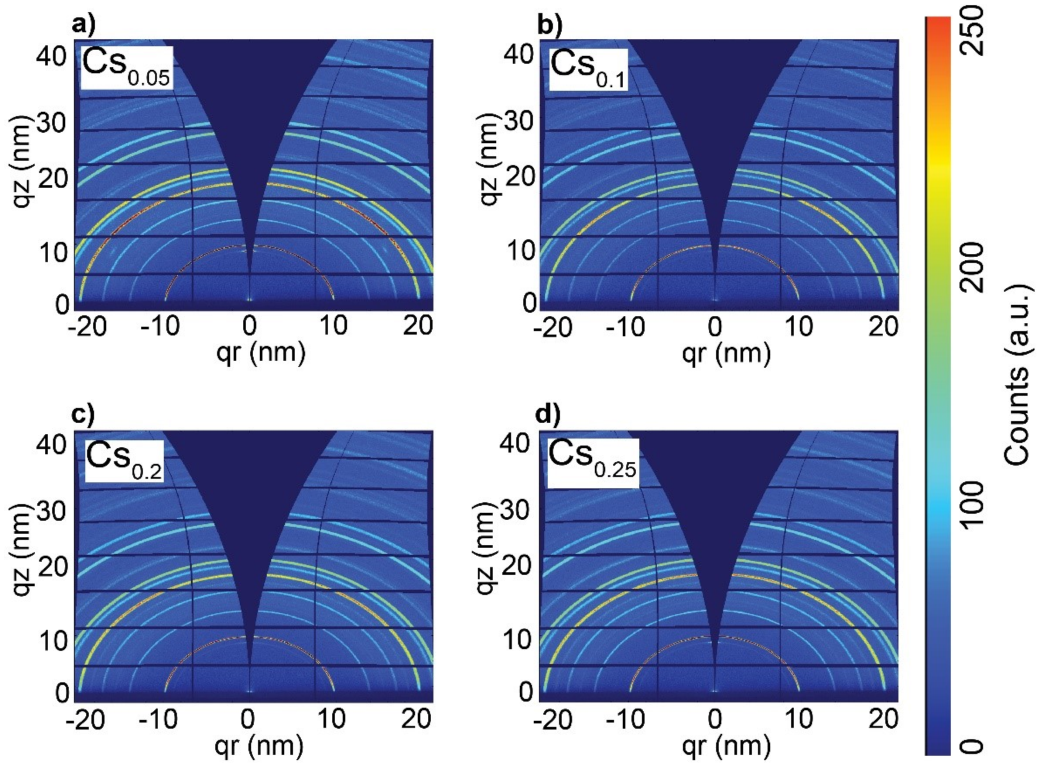

Supplementary Figure S17. a-e) 2D GIWAXS patterns of the CsFA perovskite films

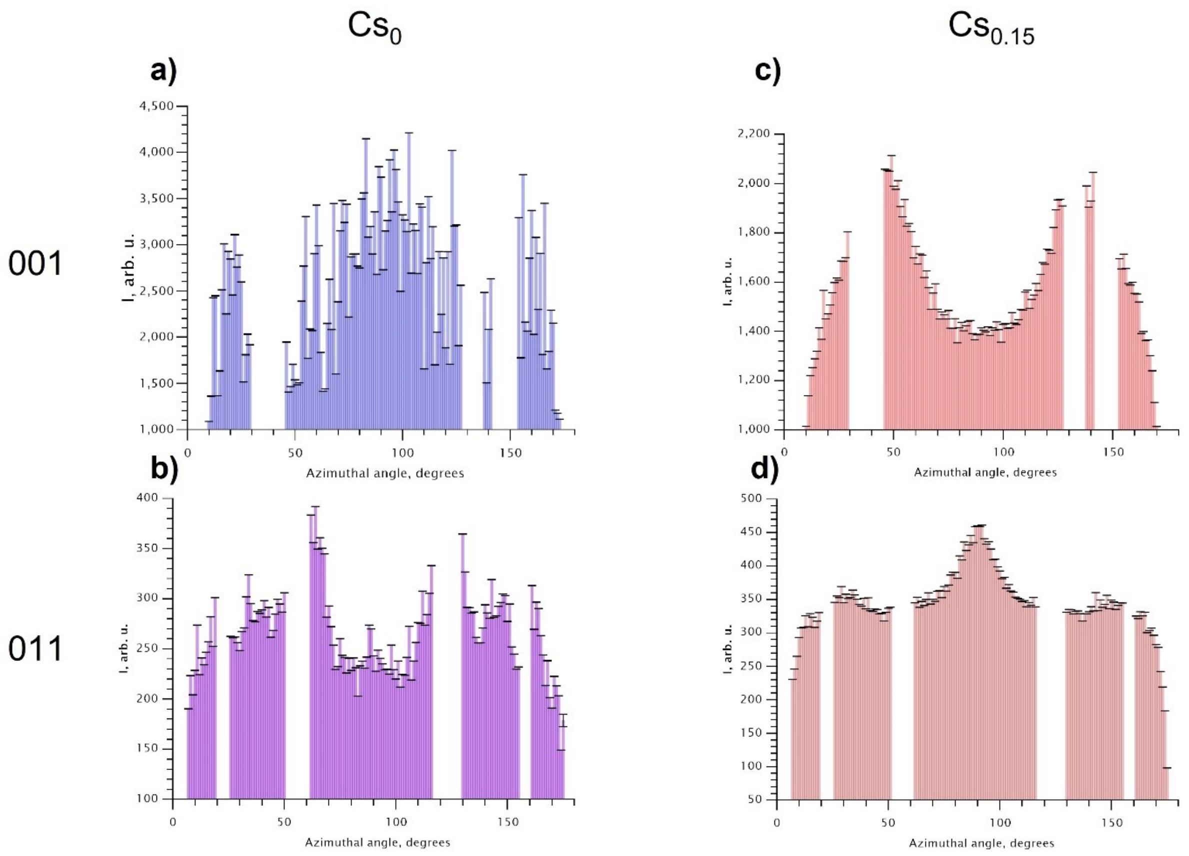

Supplementary Figure S18. Azimuthal integration of the 001 and 011 peaks, respectively: a-b) Cs_0 and c-d) $Cs_{0.15}$ perovskite films.

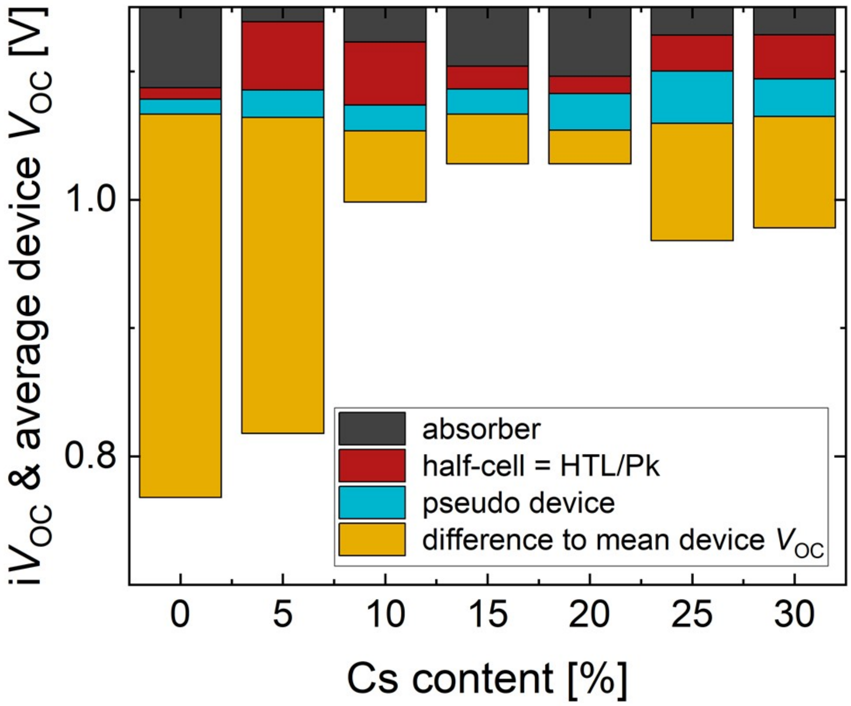

Supplementary Figure S19. Implied V_{OC} values extracted from PLQY measurements at 1 sun conditions of the different device layers for all the CsFA perovskite films. "Absorber" denotes a layout of glass/perovskite, Half-cell: ITO/MeO-2PACz/perovskite, Pseudo-device: ITO/MeO-2PACz/perovskite/C60/SnO_2 and Full device: ITO/MeO-2PACz/perovskite/C60/SnO_2/Ag (V_{OC} from *JV* measurements).

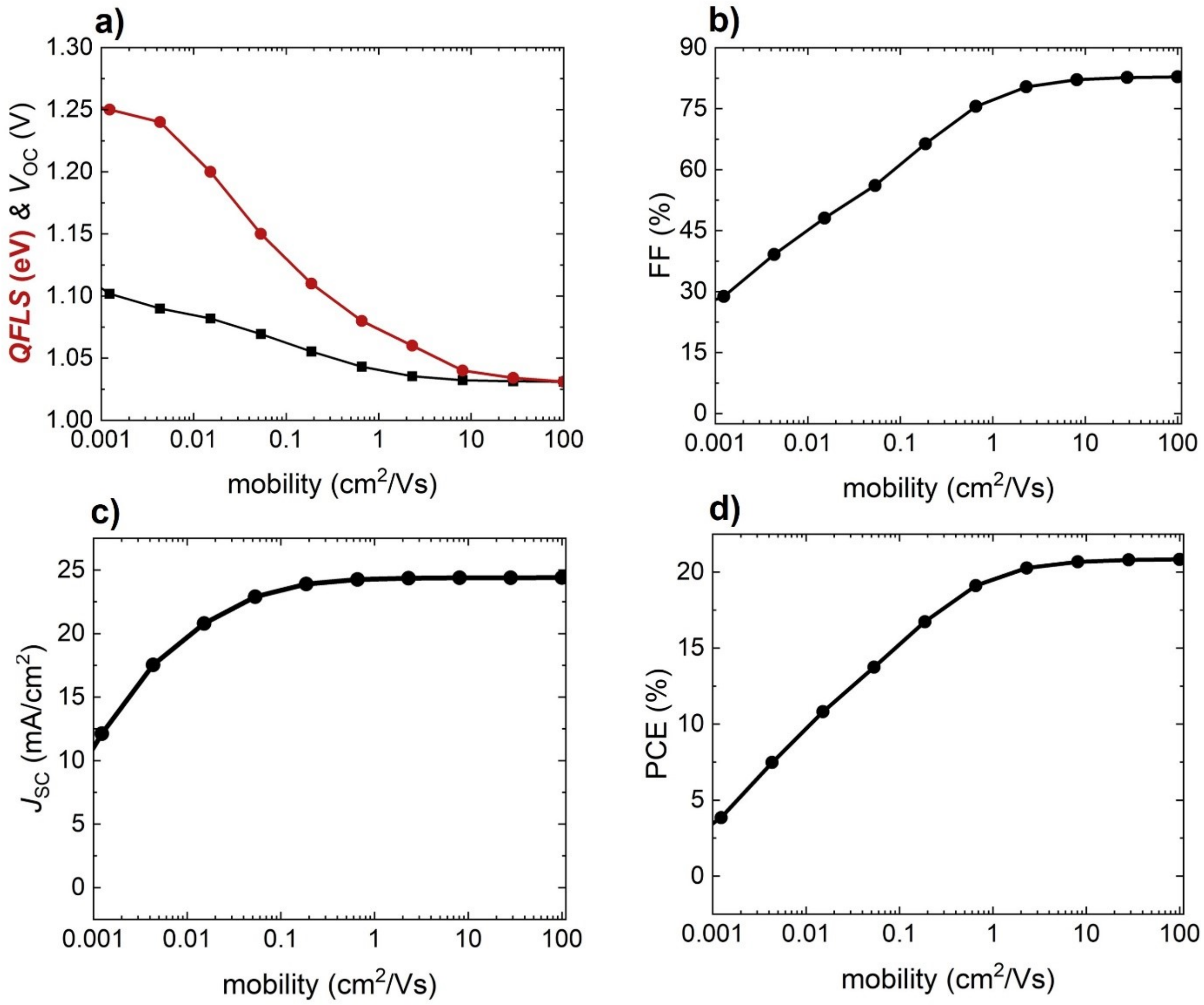

Supplementary Figure S20. a-d) Drift-diffusion simulations highlighting the device parameters and QFLS with respect to the change in carriers' mobility. Reference file available at: https://zenodo.org/records/10698870

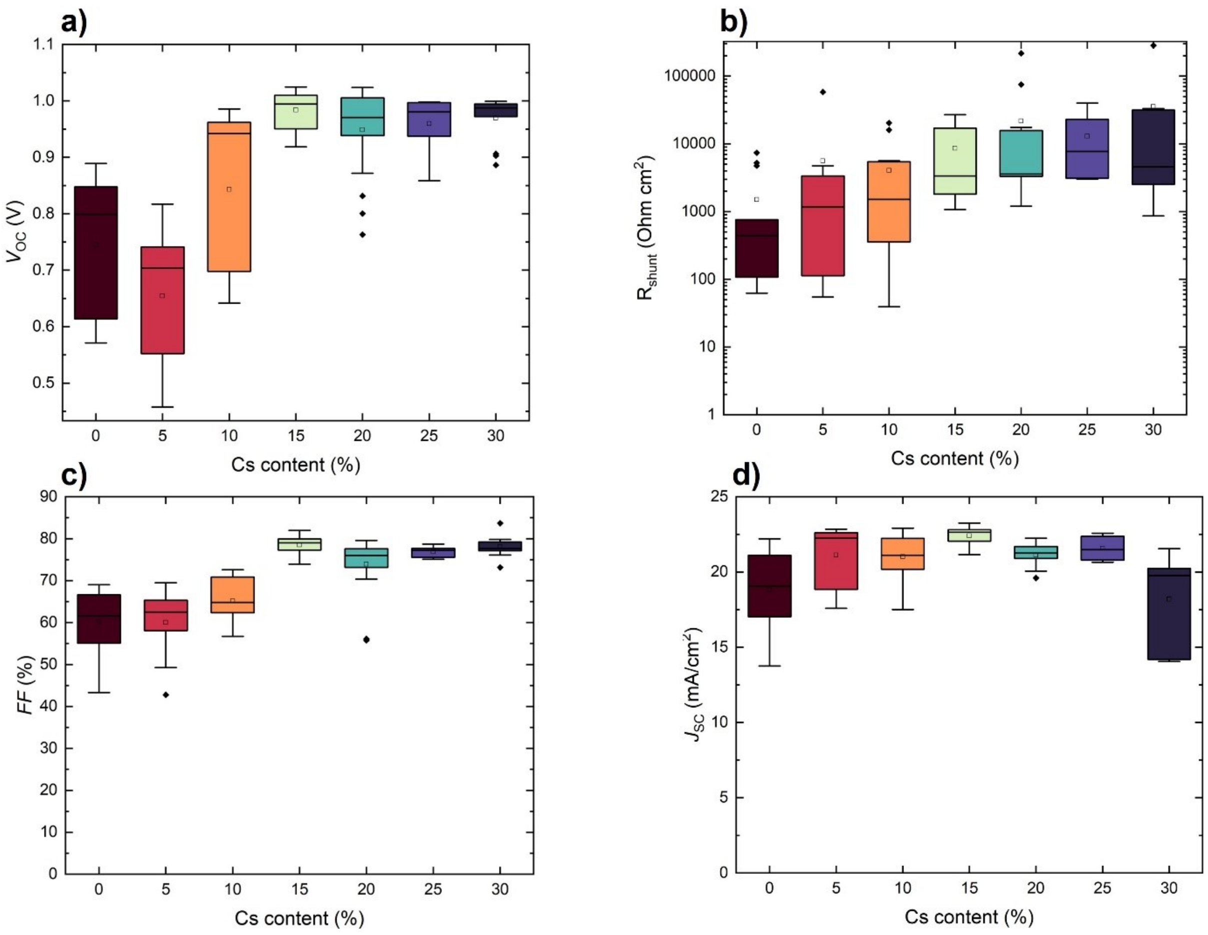

Supplementary Figure S21. a-d) Device performance metrics and calculated shunt resistances as a function of different Cs content under one sun illumination.

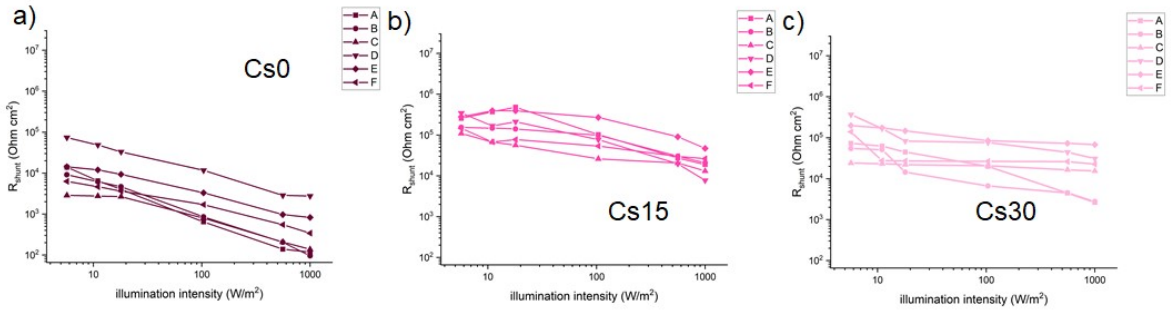

Supplementary Figure S22. a-c) Apparent shunt resistance (for 6 devices each: Cs_0, $Cs_{0.15}$, $Cs_{0.3}$) as extracted by a linear fit to the low voltage region of the JV curve (-0.05 to 0.05 V) obtained from slow *JV* scans to allow for relaxation of ions.

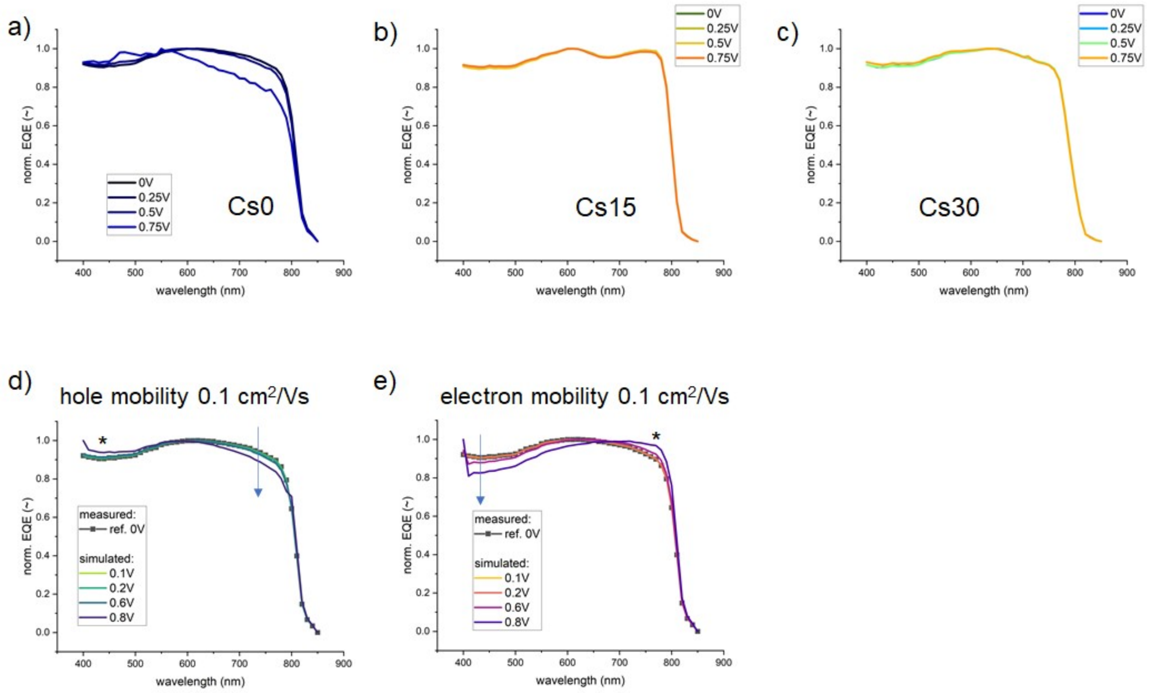

Supplementary Figure S23. a-c) Voltage-dependent normalized EQE measurements for Cs0, Cs15, Cs30. In the case of Cs0 we observe a change in the shape of the EQE with a reduction in the red part of the spectrum, absent in samples with Cs. By comparing these results to analogous drift-diffusion measurements (note: the EQEs simulated in SCAPS-1D were divided by the 0 V case to obtain a "scaling" spectrum which was then multiplied with the 0 V measurement from panel **a** for better comparability) with artificially reduced hole (**d**) or electron mobility (**e**), we conclude that reduces hole transport is the origin of the discussed losses. * denotes an artificially enhanced EQE due to normalization.

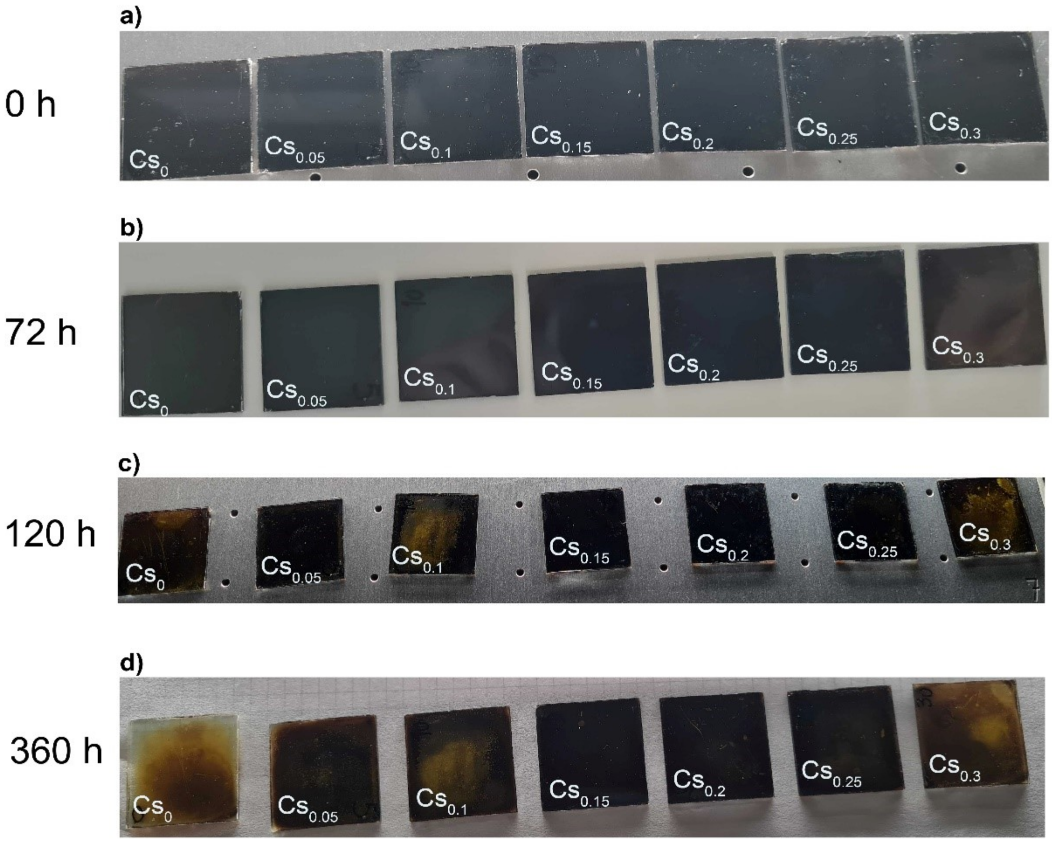

Supplementary Figure S24. a-d) Photographs of the bare $Cs_xFA_{1-x}PbI_3$ perovskite absorbers taken at different time intervals.

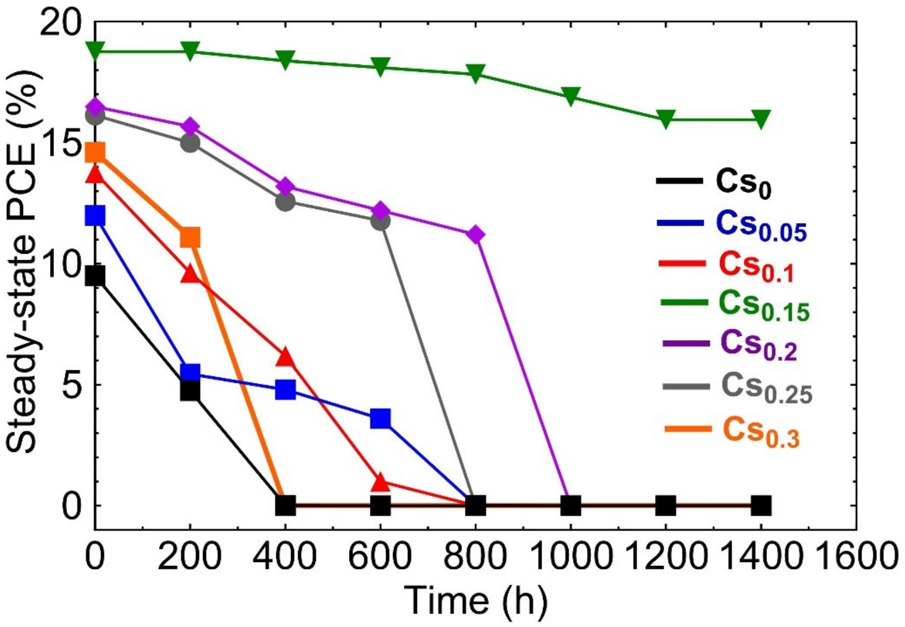

Supplementary Figure S25. Un-normalized steady-state PCEs for the encapsulated PSCs.

Table S1. Goldschmidt tolerance factor (*t*) values of the $Cs_xFA_{1-x}PbI_3$ perovskite films. The ionic radii calculations are based on ref.[2]

X in $Cs_xFA_{1-x}PbI_3$	*t* values
0	1.04
0.05	1.03
0.1	1.02
0.15	1.01
0.2	1
0.25	0.99
0.3	0.98

Table S2. Urbach energy at room temperature (RT) as obtained from apparent Urbach energy spectrum, Einstein sold model fit parameters E_0 and σ_0, and calculated approximate Einstein temperature, Θ_E, and wavenumber for the four $Cs_xFA_{1-x}PbI_3$ perovskite systems investigated by temperature dependent EQE measurements.

X in $Cs_xFA_{1-x}PbI_3$	E_U (RT) [meV]	E_0 [meV]	σ_0	Θ_E [K]	λ^{-1} [cm^{-1}]
0	12.1 ± 1	3.26 ± 0.24	2.15 ± 0.01	162	113
0.05	11.5 ± 1	3.82 ± 0.09	2.29 ± 0.01	203	142
0.1	11.8 ± 1	3.75 ± 0.15	2.20 ± 0.01	192	133
0.15	11.0 ± 1	4.55 ± 0.18	2.45 ± 0.02	259	180
0.2	11.5 ± 1	5.39 ± 0.09	2.41 ± 0.01	301	210
0.25	11.7 ± 1	6.68 ± 0.13	2.49 ± 0.02	381	266
0.3	11.5 ± 1	4.00 ± 0.15	2.30 ± 0.01	213	149

1. Hodge, I. M., Ingram, M. D. & West, A. R. Impedance and modulus spectroscopy of polycrystalline solid electrolytes. *J. Electroanal. Chem. Interfacial Electrochem.* **74**, 125–143 (1976).

2. An, Y. *et al.* Structural Stability of Formamidinium- and Cesium-Based Halide Perovskites. *ACS Energy Lett.* **6**, 1942–1969 (2021).
